# Fluorescent Molecular Rotors Based on Hinged Anthracene Carboxyimides

**DOI:** 10.3390/molecules28073217

**Published:** 2023-04-04

**Authors:** Yanhai Ni, Wangjian Fang, Mark A. Olson

**Affiliations:** 1 School of Pharmaceutical Science and Technology, Tianjin University, Tianjin 300072, China; 2 Department of Physical and Environmental Sciences, Texas A&M University Corpus Christi, Corpus Christi, TX 78412, USA

**Keywords:** anthracene carboxyimide, molecular rotor, fluorescence sensor

## Abstract

Temperature and viscosity are essential parameters in medicine, environmental science, smart materials, and biology. However, few fluorescent sensor publications mention the direct relationship between temperature and viscosity. Three anthracene carboxyimide-based fluorescent molecular rotors, **1DiAC**∙Cl, **2DiAC**∙Cl, and **9DiAC**∙Cl, were designed and synthesized. Their photophysical properties were studied in various solvents, such as N, N-dimethylacetamide, N, N-dimethylformamide, 1-propanol, ethanol, dimethyl sulfoxide, methanol, and water. Solvent polarizability resulted in a solvatochromism effect for all three rotors and their absorption and emission spectra were analyzed via the Lippert–Mataga equation and multilinear analysis using Kamlet–Taft and Catalán parameters. The rotors exhibited red-shifted absorption and emission bands in solution on account of differences in their torsion angle. The three rotors demonstrated strong fluorescence in a high-viscosity environment due to restricted intramolecular rotation. Investigations carried out under varying ratios of water to glycerol were explored to probe the viscosity-based changes in their optical properties. A good linear correlation between the logarithms of fluorescence intensity and solution viscosity for two rotors, namely **2DiAC**∙Cl and **9DiAC**∙Cl, was observed as the percentage of glycerol increased. Excellent exponential regression between the viscosity-related temperature and emission intensity was observed for all three investigated rotors.

## 1. Introduction

Solvatochromism describes a phenomenon in which a molecule exhibits different visible colors or fluorescence emissions in different solvents. This effect occurs due to alterations in the solvation power, which depends on the polarity, viscosity, intensity, and polarizability of the solvent. As such, solvatochromism is a highly complex phenomenon and is always a result of several influencing factors. The fluorescence emission of a particular fluorophore is more sensitive to solvatochromic effects than its ground-state visible color in solution because the surrounding environment simultaneously governs both the excited and the lowest energy states [1]. The Lippert–Mataga theory has been used to explain the solvent effect on the spectral shifts via the relationship between the experimental Stokes shift and the solvent polarity parameter, namely, orientation polarizability (Δ*f*) [2,3]. The Lippert–Mataga plots demonstrate a direct connection between the Stokes shift and the physical characteristics of solvents, such as dielectric constant, refractive index, and variations in dipole moment. However, some specific solvent–fluorophore interactions can affect the linear Lippert–Mataga plot, such as acid-base effects and hydrogen bonding [4,5]. Kamlet and Taft et al. [6,7,8,9] proposed the solvatochromic model to develop a greater understanding of solvatochromism. Solvent acidity, basicity, and polarity are considered the prime influencers on the effects of solvatochromism [9]. More recently, Catalán et al. [10,11,12,13] proposed another generalized model to study solvatochromism. In this case, solvent polarity parameters were further subdivided into two different parameters, namely, solvent polarizability and dipolarity [13], in an effort to obtain the solvent polarity parameter which contributes the most to solvatochromism.

Fluorescent molecular rotors (FMRs), which are typically comprised of systems bearing donor-π-acceptor (D-π-A) structures, are strong candidates for applications as microenvironment probes in membrane chemistry [14,15,16], biological systems [17,18], materials science [19,20], and as molecular thermometers [21]. Naturally, the rotation of FMRs is suppressed in high-viscosity environments, at which point the non-radiative decay of FMRs is inhibited, triggering fluorescence emission enhancement. Thus, parameters such as fluorescence quantum yields and the fluorescent lifetimes of FMRs are a priori connected to the viscosity of the surrounding environment. As viscosity is sensitive to the surrounding temperature, so are FMRs, which can easily be developed into fluorescent probes capable of monitoring both temperature and viscosity. However, in this context, few publications have mentioned the direct relationship between temperature and viscosity [22,23].

**Figure 1:**
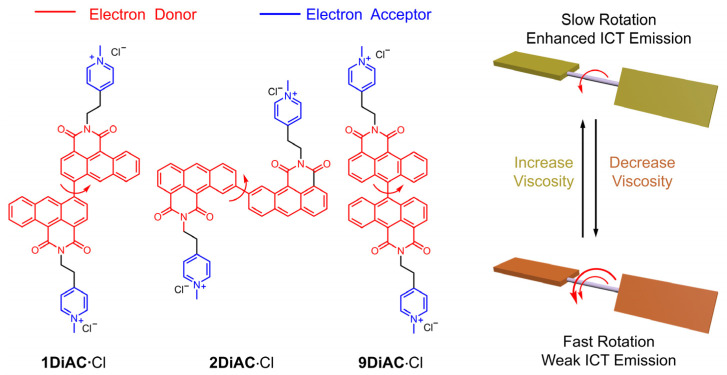
Molecular structures and sensing mechanism of the three title anthracene carboxyimide-based fluorescent molecular rotors. The number of red arrows indicates the rotation speed.

Anthracene carboxyimide, a π-acceptor (π-A) system, [24,25] demonstrates excellent emissive properties [26], and has been proven to be suitable for potential applications as antitumor agents [27], dyes [28], sensors [29], cell-imaging agents [30], and cell photodynamic therapeutics [31]. Herein, we report the synthesis of three tandem bisanthracene carboxyimide fluorescent organic salts as FMRs to develop new fluorescent sensors showing a direct relationship between temperature and viscosity. Structurally, the two anthracene carboxyimide molecules are bonded via a single bond at different positions of anthracene carboxyimide (Figure 1). Pyridine was methylated to generate a bis-pyridinium salt in order to improve solubility across different solvents. In solution, each anthracene caroboxyimide unit is an independent emitter, leading to short wavelength fluorescence. The intramolecular charge transfer among the three rotors was enhanced with increases in viscosity. The photophysical properties of the three FMRs were investigated in solution and in the solid state. Solvatochromism of the three anthracene carboxyimide-based FMRs was analyzed using Lippert–Mataga plots, the Kamlet–Taft equation, and the Catalán model. Furthermore, these FMRs were gauged for their potential to behave as molecular viscometers and viscosity-related thermometers. The relationship between fluorescence intensity and solution viscosity with different mixed ratios of water and glycerol was studied. Finally, the emission spectra of the three FMRs in glycerol solutions were also measured at different temperatures.

## 2. Results and Discussion

### 2.1. Design and Syntheses

The synthetic routes to **1DiAC**∙Cl, **2DiAC**∙Cl, and **9DiAC**∙Cl are shown in Schemes S1 to S3, respectively. They have been fully structurally characterized using ^1^H NMR, ^13^C NMR, and high-resolution mass spectrometry (HRMS). Their corresponding hexafluorophosphate salts, **1DiAC**∙PF_6_, **2DiAC**∙PF_6_, and **9DiAC**∙PF_6_, were obtained via counterion exchange, in order to measure their ^1^H NMR spectra and to grow crystals suitable for X-ray crystallographic analysis. For the most part, the synthetic routes to **1DiAC**∙Cl and **2DiAC**∙Cl are the same. Generally, 1-bromoanthracene or 2-bromoanthracene, oxalyl chloride, and aluminum chloride were stirred at room temperature for 24 h in order to undergo the Friedel–Crafts acylation reaction and yield the corresponding anthracene diones, namely, **1An Dione** or **2An Dione**. The anthracene diones were then oxidized with hydrogen peroxide to obtain anthracene anhydrides, namely, **1An Anhydride** or **2An Anhydride**. Then, a reaction mixture of anthracene anhydride and 4-(2-aminoethyl)pyridine in ethanol underwent nucleophilic addition elimination reactions to give anthracene carboxyimide species, **1AC** or **2AC**. In the following step, bisanthracene carboxyimide molecules, **1DiAC** or **2DiAC**, were obtained via the Miyaura borylation reaction and Suzuki–Miyaura coupling. Finally, the reaction mixture of bisanthracene carboxyimide, **1AC** or **2AC**, and iodomethane in N, N-dimethylformamide was subjected to the Menshutkin reaction and subsequently counterion exchange to give the final products, **1DiAC**∙Cl or **2DiAC**∙Cl. The synthetic route of **9DiAC**∙Cl was similar to that of **1DiAC**∙Cl and **2DiAC**∙Cl, with the only exception being that the already fused starting material, 9,9′-bianthracene, was used.

### 2.2. Photophysical Properties and Solvatochromism

**Figure 2:**
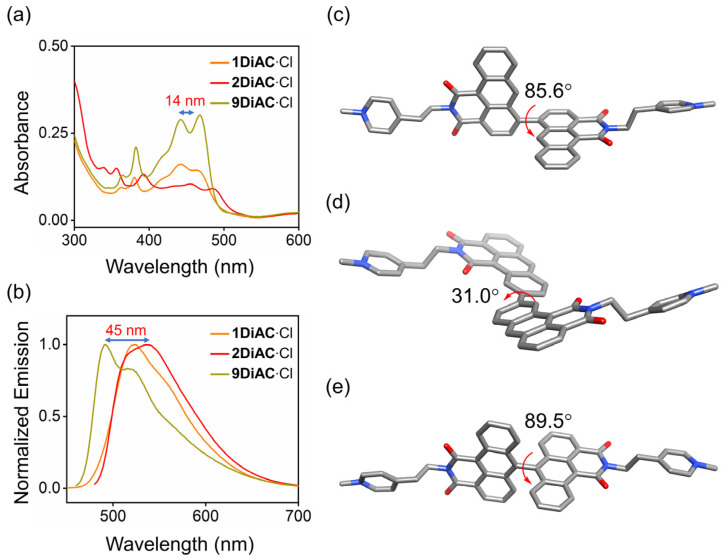
(**a**) Overlayed absorption and (**b**) normalized emission spectra of **1DiAC**·Cl, **2DiAC**·Cl, and **9DiAC**·Cl in DMA. c = 1 × 10^−5^ M. Gas phase optimized geometries of (**c**) **1DiAC**∙Cl, (**d**) **2DiAC**∙Cl, and (**e**) **9DiAC**∙Cl at a dielectric constant of ε_r_ = 1.00 and their corresponding dihedral angles between the fused anthracene carboxyimide moieties using the B3LYP functional with a 6–31G(d,p) basis set. Hydrogen atoms and counterions have been omitted for clarity.

The absorption and fluorescence emission properties of all three molecular rotors, **1DiAC**·Cl, **2DiAC**·Cl, and **9DiAC**·Cl, were studied in a total of seven solvents, namely N, N-dimethylacetamide (DMA), N, N-dimethylformamide (DMF), 1-propanol (1-PrOH), ethanol (EtOH), dimethyl sulfoxide (DMSO), methanol (CH_3_OH), and water (H_2_O) (Figures S1 and S2). The photophysical properties of all three compounds in these solvents are summarized in Table S1. As shown in Figure 2a,b, rotors **9DiAC**·Cl, **1DiAC**·Cl, and **2DiAC**·Cl produced red-shifted absorption (442 = 442 < 456) and emission (491 < 522 < 536) spectra in DMA. The optimized structures of the three rotors were modeled to further investigate the apparent red shift in their absorption and emission spectra by density functional calculations in a dielectric medium of ε_r_ = 1.00 at the B3LYP/6–31G(d, p) level. The optimized structures of all three rotors in the ground state are shown in Figure 2c–e, respectively. The dihedral angles between the two fused anthracene carboxyimide moieties in **1DiAC**·Cl, **2DiAC**·Cl, and **9DiAC**·Cl are 85.6, 31, and 89.5°, respectively. The degree of coplanarity of the three rotors was thus ordered as **2DiAC**·Cl > **1DiAC**·Cl > **9DiAC**·Cl. As the degree of coplanarity increased (smaller dihedral angle), the aromatic π-system of the donor component of the rotors became more extended, resulting in a red-shifted absorption and emission spectra. Single crystals suitable for X-ray crystallographic analysis of **9DiAC**∙PF_6_ were obtained via slow evaporation from dichloromethane and acetonitrile (Figure S9). The dihedral angle between the two anthracene carboxyimide moieties was observed to be 83.5°, which was similar in value to its calculated optimized structure.

**Figure 3:**
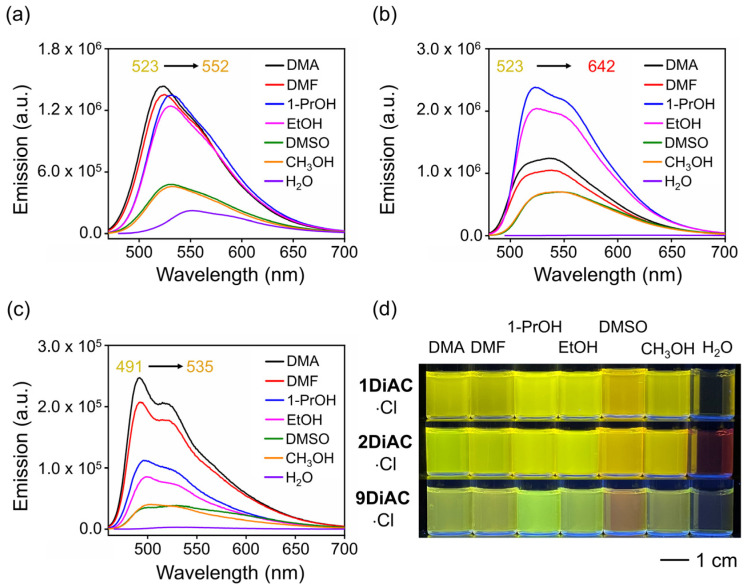
Emission spectra of (**a**) **1DiAC**·Cl, (**b**) **2DiAC**·Cl, and (**c**) **9DiAC**·Cl in different solvents. (**d**) Photograph of vials containing **1DiAC**·Cl, **2DiAC**·Cl, and **9DiAC**·Cl in N, N-dimethylacetamide (DMA), N, N-dimethylformamide (DMF), 1-propanol (1-PrOH), ethanol (EtOH), dimethyl sulfoxide (DMSO), methanol (CH_3_OH), and water (H_2_O) under 365 nm light depicting fluorescence emission solvatochromic changes.

The emission spectra for the rotors were found to be strongly solvent-dependent (Figure S2 and Figure 3d), whereas only minor changes in their absorption spectra were observed (Figure S1). Two absorption regions were recorded for each of the three rotors, namely a π-π* transition region from 260 to 300 nm and an intramolecular charge transfer (ICT) band from 350 to 500 nm (Figure S1a–c). **1DiAC**·Cl, **2DiAC**·Cl, and **9DiAC**·Cl produced absorption bands in the ranges of 442–475, 456–489, and 442–458 nm, respectively, in various solvents. Compared with **1DiAC**·Cl and **9DiAC**·Cl, **2DiAC**·Cl showed an apparent solvatochromic change under visible light (Figure S1d). In addition, the fluorescence emission of the three rotors, **1DiAC**·Cl, **2DiAC**·Cl, and **9DiAC**·Cl, was observed in the ranges of 522–552, 523–642, and 491–535 nm, respectively, for the different solvents (Figure 3a–c). The three rotors underwent fluorescence quenching as a result of an increase in non-radiative processes as the solvent polarity was increased. Solvatochromic shifts were also observed with increases in the solvent polarity, indicating that the excited state of the three FMRs is sensitive to solvent polarity. To further study the photophysical properties of the three FMRs, the absorption and emission spectra of a derivative bearing a single anthracene carboxyimide group, **2AC**, were measured in the same solvents (Figure S3 and Table S2). The emission spectra of **2AC** indicated that upon fusing the anthracene carboxyimide groups to one another, red shifts for both the absorption and emission spectra were evident when compared to **2AC**, the extent of which was augmented as the degree of coplanarity increased. Thus, **9DiAC**·Cl is very similar to **2AC**, as it possesses the highest dihedral angle and is the least coplanar. Moreover, the data also suggested that each anthracene carboxyimide unit is an independent emitter.

**Figure 4:**
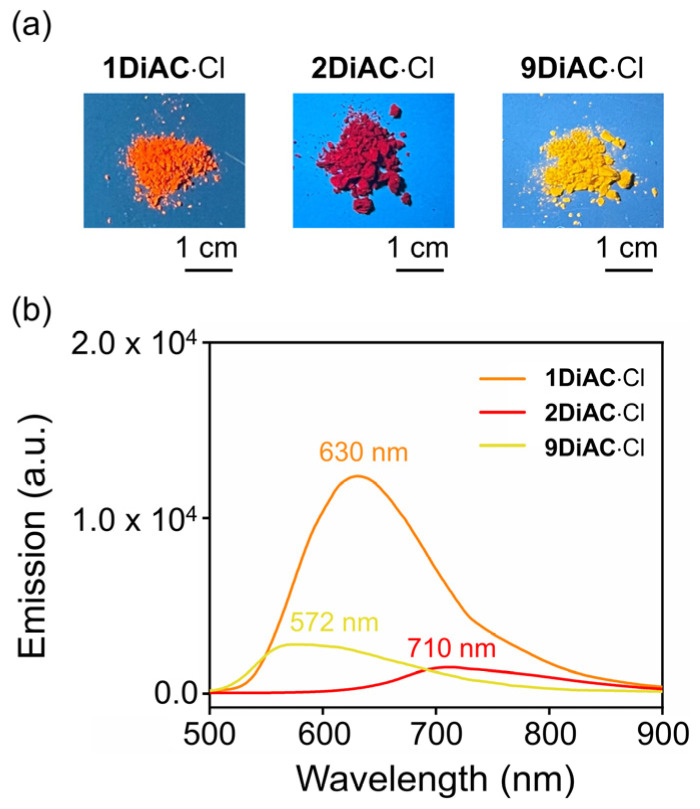
(**a**) Photographs of **1DiAC**∙Cl, **2DiAC**∙Cl, and **9DiAC**∙Cl in solid state, illustrating the color of their fluorescence emission under excitation at 365 nm. (**b**) Solid-state emission spectra of **1DiAC**∙Cl, **2DiAC**∙Cl, and **9DiAC**∙Cl at room temperature. Excitation at 365 nm.

All photophysical parameters of the three FMRs, such as Stokes shift, absolute fluorescent quantum yield, and fluorescence lifetime, were studied in seven solvents with differing polarities. The FMRs each demonstrated that the absolute fluorescent quantum yield was weakened with the increases in solvent polarity. No apparent relationship between fluorescence lifetime and solvent polarity was observed for the three FMRs. In the solid state, **1DiAC**·Cl, **2DiAC**·Cl, and **9DiAC**·Cl produced broad emission bands at 630, 710, and 572 nm when excited at 365 nm, respectively (Figure 4). The emission of the rotors proved to be more red-shifted in the solid state than in the solution.

The solvatochromic phenomenon of the three FMRs was first explored in the context of the Lippert–Mataga theory [2,3]. The Lippert–Mataga equation between Stokes shift and orientation polarity (Δ*f*) is shown in Equation (1):

(1) $$\Delta \nu =\nu_{A}-\nu_{F}=\frac{2}{\mathrm{hc}}(\Delta f)\frac{{\left(\mu_{e}-\mu_{g}\right)}^{2}}{a^{3}}+C$$

where *ν_A_* and *ν*_F_ are the wavenumbers (cm^−1^) of the absorption and emission center, respectively, h is the Planck’s constant (6.626 × 10^−34^ J·s), c is the speed of light (2.9979 × 10^8^ m/s), *a* is the Onsager radius, and Δ*μ* = *μ_e_* − *μ_g_* is the difference in dipole moment between ground and excited states. Orientation polarizability (Δ*f*) is calculated using Equation (2):

(2) $$\Delta f=f(\varepsilon )-f(n^{2})=\frac{\varepsilon -1}{2\varepsilon +1}-\frac{n^{2}-1}{2n^{2}+1}$$

where *ɛ* is the solvent dielectric constant, and *n* is the solvent refractive index.

General solvent effects are represented as a spectral shift when a good linear Lippert–Mataga plot is obtained. If not, specific solute–solvent interactions dominate and drive the spectral shift. The Lippert–Mataga plots of the Stokes shift and orientation polarizability for all three of the molecular rotors are shown in Figure S4. Linear correlation plots for the three FMRs indicated the presence of strong intramolecular charge transfer (ICT) for their excited state [32]. The negative slope of the fitting curve for **1DiAC**∙Cl and the poor linear behavior of **9DiAC**∙Cl revealed that specific solute–solvent interactions control the spectral shift for these two rotors. However, the Lippert–Mataga plots of **2DiAC**∙Cl proved to be linear with a regression coefficient (R^2^) of 0.888, which indicated that the solvent parameters, namely the dielectric constant and refractive index, are responsible for the recorded spectral red shift.

### 2.3. Multilinear Analysis Using Kamlet–Taft and Catalán Parameters

The data informed us that the Lippert–Mataga theory could not precisely explain the observed solvatochromism. Hence, the multi-parameter approach proposed by Kamlet–Taft [6,7,8,9] and Catalán [10,11,12,13] was employed in order to further study and explain the solvatochromism. These two multi-parameter methods have been successfully applied to various physiochemical processes, working with, for example, UV–Vis absorption centers [33], emission centers [33], Stokes shifts [33], quantum yields [34], radiative and non-radiative rate constants [34], and fluorescent lifetimes [34]. Kamlet–Taft and Catalán solvatochromic equations are expressed as Equations (3) and (4), respectively:

(3) $$y=y_{0}+a_{\alpha }\alpha +b_{\beta }\beta +c_{\pi *}\pi *$$

where *y* represents a solvent-affected physiochemical characteristic, such as UV–Vis absorption center (*ν*_Abs_), emission center (*ν*_Em_), and Stokes shift (Δ*ν*_St_), *y*_0_ is the observed characteristic, *a_α_*, *b_β_*, and c_π*_ are the coefficients that show the relationship between *y* and various solvent properties, *α* is the solvent acidity, *β* is the solvent basicity, π* is the coefficient of solvent polarity, polarizability, and dipolarity.

(4) $$y=y_{0}+a_{SA}SA+b_{SB}SB+c_{SP}SP+d_{SDP}SDP$$

where *y* is a solvent-affected physiochemical characteristic, such as UV–Vis absorption center (*ν*_Abs_), emission center (*ν*_Em_), and Stokes shift (Δ*ν*_St_), *y*_0_ is the observed characteristic, *a_SA_*, *b_SB_*, *c_SP_*, and *d_SDP_* are the coefficients that show the relation between *y* and various solvent properties, *SA* is the solvent acidity, *SB* is the solvent basicity, *SP* is the solvent polarizability, *SDP* is the solvent dipolarity.

The Kamlet–Taft and Catalán parameters are used to determine the main contributing feature which drives the observed changes in absorption spectra, emission spectra, Stokes shifts, and absolute quantum yield in various solvents. The positive and negative values for specific parameters in the multilinear analysis method suggested negative and positive solvatochromism, respectively. The solvent parameters of the Kamlet–Taft and Catalán equations are summarized in Table S4. In addition, the detailed results obtained by combining the Kamlet–Taft and Catalán equations for the three FMRs in a series of solvents are shown in Tables S5–S7, respectively. The multilinear analysis demonstrated that Catalán regression coefficients are better than Kamlet–Taft correlation coefficients for explaining the obtained results.

Negative solvatochromism was observed for the three FMRs in their absorption and emission spectra. According to the results for **1DiAC**∙Cl, the high regression value of the Catalán parameters (0.962) for solvent polarizability (*c_SP_*) was principally responsible for the observed slight red shift in the absorption spectra. In addition, a high regression value was obtained for Catalán parameters (0.958), which implies that solvent polarizability (*c_SP_*) is mainly responsible for the observed red shift in common solvents. Solvent dipolarity (*d_SDP_*) controls the changes in absolute quantum yield with a 0.82 regression value of the Catalán parameters. In the case of **2DiAC**∙Cl, high regression values of 0.956, 0.990, 0.996, and 0.96 were obtained for *ν*_Abs_, *ν*_Em_, Δ*ν*_St_, and Φ_F_, respectively. Solvent dipolarity is the main driver for changes in the absorption spectra, whereas solvent polarizability and basicity also significantly affect the red shift in the absorption spectra. Solvent polarizability also plays a prominent role in causing the red shift in the fluorescence emission spectra. Changes in solvent dipolarity causes the changes in absolute quantum yield observed in various solvents. Based on the multilinear analysis of **9DiAC**∙Cl, good multilinear fits of 0.991, 0.997, 0.976, and 0.74 were observed for *ν*_Abs_, *ν*_Em_, Δ*ν*_St_, and Φ**_F_**, respectively. Thus, solvent polarizability appeared to be the dominant factor in the alteration of the absorption and emission spectra of the synthesized FMRs, while solvent dipolarity is responsible for changes in absolute quantum yield.

### 2.4. Viscosity Response of the Fluorescent Molecular Rotors

**Figure 5:**
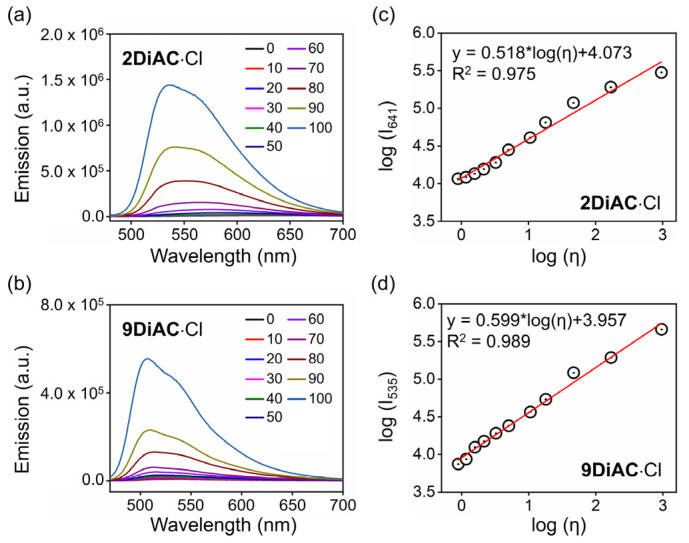
Emission spectra of (**a**) **2DiAC**·Cl and (**b**) **9DiAC**·Cl recorded in mixtures of water and glycerol at different ratios of increasing glycerol content from 0 to 100%. The linear response between log (I_x_) (I_x_ is the emission intensity at the emission center of (**c**) **2DiAC**·Cl and (**d**) **9DiAC**·Cl in aqueous solution) and log (viscosity) in mixtures between water and glycerol at different ratios. c = 1 × 10^−5^ M.

It is well-known that the rate of a non-radiative deactivation process can be influenced by solvent viscosity due to the intramolecular rotation in a fluorophore [35]. With exception to solvatochromism, the three FMRs responded strongly to changes in solvent viscosity. An evident blue shift of the UV–Vis absorption spectra of **1DiAC**·Cl and **2DiAC**·Cl was observed in mixtures of various ratios of water and glycerol (Figure S5a,b). However, there were no obvious changes in the UV–Vis absorption spectra of **9DiAC**·Cl (Figure S5c). Unlike UV–Vis absorption, the apparent blue shift and fluorescence emission enhancement were observed for all three FMRs with an increase in glycerol percentage. These changes appeared concomitantly with changes in solution viscosity and polarity (Figure 5a,b and Figure S6). Such viscosity-dependent fluorescence emission enhancement is due to the rotation restriction between two anthracene carboxyimide rings [36]. As shown in Figure 5c,d, the logarithm of the fluorescent intensity of **2DiAC**·Cl and **9DiAC**·Cl as a function of the logarithm of solution viscosity (Table S8) showed good linear behavior according to the Förster–Hoffmann Equation (Equation (5)):

(5) logI=C+xlogη

where *I* is the emission intensity of the aqueous solution emission center, *C* is the constant, *x* is the viscosity sensitivity, and *η* is viscosity in mPa∙s.

The slopes of the **2DiAC**·Cl and **9DiAC**·Cl fitting lines based on the Förster–Hoffmann equation were 0.518 and 0.599, respectively, which proved to be higher in sensitivity compared with previous work [14]. As the glycerol percentage increased, fluorescence emission enhancement appeared first for **1DiAC**·Cl. Fluorescence quenching and blue shifting of the spectra then followed when the glycerol ratio increased to 50%. A blue shift in emission was observed when the glycerol ratio reached 60%. Augmentation of the fluorescence emission intensity occurred when the glycerol ratio was further increased to 80%, due to the restricted intramolecular rotation of the anthracene carboxyimide moieties at the higher viscosity.

To further study the blue shift observed for the emission spectra of all the rotors, an 80% glycerol aqueous solution was selected because the viscosity of an 80% glycerol-water mixture is about 5.08 mPa·s at 80 °C. This is a value which is very close to the viscosity value of a 50% glycerol-water mixture at 25 °C, namely 5.04 mPa·s. The emission spectra of all three rotors in the 80% glycerol-water mixture was measured at 25 and 80 °C (Figure S7). Only fluorescence quenching was observed for all rotors at 80 °C because of the non-radiative decay of the rotors at high temperatures. This result revealed that solvent polarity was responsible for the blue shift in the emission spectra of the three rotors when the percentage of glycerol was increased.

### 2.5. Viscosity-Related Temperature Response of the Fluorescent Molecular Rotors

**Figure 6:**
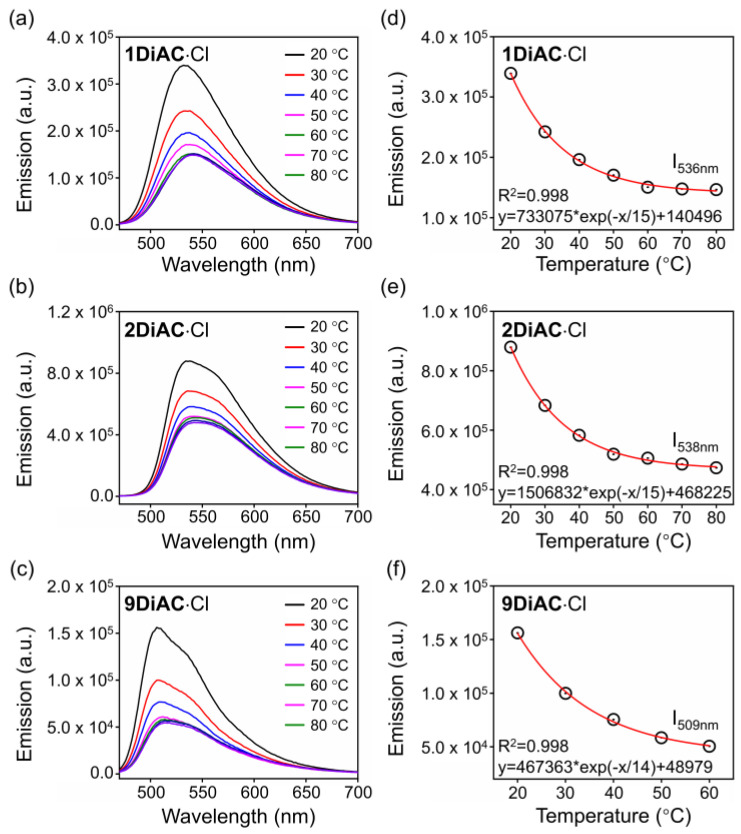
Emission spectra of (**a**) **1DiAC**·Cl, (**b**) **2DiAC**·Cl, and (**c**) **9DiAC**·Cl measured in glycerol from 20 to 80 °C. Temperature dependence of the fluorescence intensity of (**d**) **1DiAC**·Cl, (**e**) **2DiAC**·Cl, and (**f**) **9DiAC**·Cl in glycerol. c = 1 × 10^−5^ M.

The surrounding temperature can influence viscosity, so the fluorescence emission spectra of the FMRs were measured at various temperatures to explore the direct connection between viscosity and temperature. As illustrated in Figure 6, fluorescence quenching was found for all FMRs as the temperature increased due to the free rotation between the two anthracene carboxyimide units. Excellent regression was found for all FMRs when monitoring the changes in fluorescence emission versus temperature. To verify whether temperature-dependent viscosity alterations were to blame, the same experiment was conducted in a DMF solution for the three rotors. The high boiling point and low vapor pressure of DMF help to insure that sample concentration remains constant during heating. Fluorescence intensity did not change for **1DiAC**·Cl and **2DiAC**·Cl; however, slight fluorescence quenching was observed for **9DiAC**·Cl, illustrating a high sensitivity toward to low viscosity region for **9DiAC**·Cl (Figure S8).

## 3. Conclusions

In conclusion, three novel anthracene carboxyimide-based fluorescent molecular rotors were successfully designed and synthesized. All three rotors demonstrated ICT characteristics in different solvents, related to solvent polarity. The solvatochromic behavior of the three rotors was analyzed using the Lippert–Mataga plot, Kamlet–Taft, and Catalán multilinear analysis. The results of the multilinear analysis showed that solvent polarizability drives the changes in the bathochromic-shifted absorption spectra of **1DiAC**·Cl and **9DiAC**·Cl, while solvent dipolarity is responsible for the red-shifted absorption spectra of **2DiAC**·Cl. With regard to fluorescence emission, solvent polarizability governed the red-shifted emission spectra for all three rotors, while solvent dipolarity was responsible for changes in the absolute quantum yield for the three rotors. **2DiAC**·Cl and **9DiAC**·Cl demonstrated good fluorescence sensitivity in water-glycerol mixtures, and thus can be used as fluorescent viscosity probes. Furthermore, the viscosity-dependent temperature fluorescence behavior was explored for the three rotors and excellent exponential regression was found in the relationship between emission intensity and viscosity-related temperature. Based on these new insights, we believe that the fused anthracene carboxyimide molecular rotor design strategy can be used to develop viscosity-dependent temperature fluorescent sensors of increasing complexity and functionality.

## 4. Experimental Section

### 4.1. General

Starting materials and reagents were purchased from Tokyo Chemical Industry (TCI, Shanghai, China) and used as received. 1-Bromoanthracene was synthesized following procedures reported in the literature [37]. Analytical thin-layer chromatography (TLC) was performed on aluminum sheets precoated with silica gel 60-F254 (Merck 5554). Deuterated solvents (Cambridge Isotope Laboratories, Shanghai, China) for NMR spectroscopic analyses were used as received. ^1^H and ^13^C NMR spectra were recorded on a Ascend 400 MHz spectrometer (Bruker, Beijing, China). Chemical shifts were reported in ppm relative to the residual signal of the solvent (CDCl_3_: δ 7.26 ppm for ^1^H and 77.16 ppm for ^13^C, CD_3_CN: δ 1.94 ppm for ^1^H and 1.32, 118.26 ppm for ^13^C, (CD_3_)_2_SO: δ 2.50 ppm for ^1^H and 39.52 ppm for ^13^C). High-resolution electrospray ionization (HR-ESI) mass spectral analyses were performed by a Q Exactive HF/UltiMate 3000 RSLCnano (Thermo Electron, Beijing, China) device. Solution UV–Vis absorption spectra were measured on a Cary 60 UV–Vis spectrometer using a cuvette with a 1 cm path length at 298 K. Fluorescence emission spectra, fluorescent lifetime, and absolute quantum yield were recorded on an FLS980 time-resolved fluorescence spectrometer (Edinburgh Instrumen, Livingston, England) equipped with an integrating sphere. Solid-state emission spectra were recorded on an QE65PRO fiber optic spectrometer (Ocean Optics, Largo, FL, USA). Single crystals suitable for X-ray crystallographic analysis were selected, and their X-ray diffraction intensity data were collected on a rotating anode diffractometer equipped with a hybrid photon-counting detector, using graphite-monochromated Cu Kα radiation (λ = 1.54184) at T = 293 K. Solution viscosity of mixtures with different water and glycerol ratio was measured using an AND SV-10 vibrating viscometer. DFT/TD-DFT calculations were carried out using the B3LYP functional with 6–31G(d,p) basis set as incorporated in the Gaussian 09 package for all atoms.

### 4.2. Syntheses

#### 4.2.1. Synthesis of 1An Dione

Oxalyl chloride (2.5 mL, 29.2 mmol) was added to a solution of 1-bromoanthracene (1 g, 3.9 mmol) in dichloromethane (15 mL) at 0 °C. AlCl_3_ (0.83 g, 6.2 mmol) was gradually added to the reaction mixture. Additional AlCl_3_ (0.62 g, 1.7 mmol) was added to the reaction mixture after 2 h. After 24 h, dilute aqueous HCl (1 M) was added, and the orange precipitate formed was separated via filtration. The filter cake was washed with water and then digested with 50 mL of 5% NaOH. The resulting solid was washed with water and air-dried to give the **1An Dione** product (0.69 g, 57%). Mp: 255 ± 0.5 °C. ^1^H NMR (400 MHz, DMSO, 25 °C) δ = 9.20 (s, 1H), 9.02 (dd, *J* = 8.6, 1.0 Hz, 1H), 8.57 (d, *J* = 8.7 Hz, 1H), 8.23 (d, *J* = 7.2 Hz, 1H), 8.02–7.96 (m, 1H), 7.95 (d, *J* = 7.2 Hz, 1H), 7.88–7.82 (m, 1H). ^13^C NMR (101 MHz, DMSO, 25 °C) δ = 121.53, 123.33, 123.99, 126.32, 126.71, 127.09, 127.92, 128.35, 130.28, 131.02, 131.21, 132.70, 133.04, 145.13, 187.15, 187.37. HRMS (ESI): *m*/*z* calcd for C_16_H_7_BrO_2_: 310.97022; found: 310.97000 [M+H]^+^.

#### 4.2.2. Synthesis of 1An Anhydride

**1An Dione** (1.5 g, 4.8 mmol) and NaOH (0.6 g, 14.4 mmol) were dissolved in a mixture of 1,4-dioxane (30 mL) and deionized water (10 mL). The reaction mixture was stirred at 90 °C for 20 min, and an aqueous hydrogen peroxide solution (20 mL, 30%) was dropped into the reaction mixture. The reaction mixture was stirred for another 5 h after adding an aqueous hydrogen peroxide solution. After cooling to room temperature, anthracene carboxy acid was precipitated by adding distilled water (100 mL) and concentrated H_2_SO_4_ (10 mL), and the resulting reaction mixture was allowed to stand for 12 h. The orange anhydride was collected by vacuum filtration and dissolved in 2N aqueous KOH. The insoluble material was separated via filtration, and pure **1An anhydride** (1.2 g, 78%) was obtained by adding concentrated HCl, collected via vacuum filtration, washed with distilled water, and air-dried without any further purification. Mp: 226 ± 1 °C. ^1^H NMR (400 MHz, DMSO, 25 °C) δ = 9.54 (dd, *J* = 9.1, 0.7 Hz, 1H), 9.41 (s, 1H), 8.54 (d, *J* = 8.5 Hz, 1H), 8.47 (d, *J* = 7.7 Hz, 1H), 8.28 (d, *J* = 7.7 Hz, 1H), 8.07–7.97 (m, 1H), 7.82 (dd, *J* = 11.5, 4.4 Hz, 1H). ^13^C NMR (101 MHz, DMSO, 25 °C) δ = 112.71, 118.92, 124.99, 127.01, 127.52, 130.36, 130.93, 131.06, 131.47, 132.47, 132.86, 132.95, 134.54, 136.90, 159.89, 160.38. HRMS (ESI): *m*/*z* calcd for C_16_H_7_BrO_3_: 326.96513; found: 326.96475 [M+H]^+^.

#### 4.2.3. Synthesis of 1AC

The mixture of **1An Anhydride** (2 g, 6.4 mmol) and 4-(2-Aminoethyl)pyridine (0.76 mL, 6.4 mmol) was dissolved in ethanol (80 mL), and the resulting reaction mixture was stirred at 90 °C for 24 h. After cooling to room temperature, the pure imide product **1AC** (2.27 g, 82%) was obtained by vacuum filtration and washed with cold ethanol. Mp: 228 ± 1 °C. ^1^H NMR (400 MHz, CDCl_3_, 25 °C) δ = 9.84 (d, *J* = 9.1 Hz, 1H), 9.05 (s, 1H), 8.54 (d, *J* = 5.7 Hz, 2H), 8.43 (d, *J* = 7.6 Hz, 1H), 8.08 (d, *J* = 8.4 Hz, 1H), 7.97 (d, *J* = 7.7 Hz, 1H), 7.87–7.79 (m, 1H), 7.68–7.60 (m, 1H), 7.33 (d, *J* = 5.8 Hz, 2H), 4.53–4.38 (m, 2H), 3.15–2.98 (m, 2H). ^13^C NMR (101 MHz, CDCl_3_, 25 °C) δ = 33.71, 40.99, 115.77, 121.87, 124.59, 126.61, 127.23, 127.40, 128.84, 129.66, 130.32, 131.61, 132.29, 132.93, 133.05, 133.75, 136.29, 148.25, 149.75, 163.03, 164.57. HRMS (ESI): *m*/*z* calcd for C_23_H_15_BrN_2_O_2_: 433.03692; found: 433.03653 [M+H]^+^.

#### 4.2.4. Synthesis of 1DiAC

A solution of bis(pinacolato)diboron (BPD) (0.59 g, 2.32 mmol) and **1AC** (1 g, 2.32 mmol) in 1,4-dioxane was Ar-bubbled for 30 min. Potassium acetate (0.45 g, 4.64 mmol) and [1,1′-Bis(diphenylphosphino)ferrocene]dichloropalladium(II) (138 mg, 0.19 mmol) were quickly added, and the reaction mixture was heated to 80 °C for 4 h under an Ar atmosphere. After checking the reaction progress relative to the amount of the starting material **1AC** by TLC, i.e., how much of **1AC** was consumed, one equivalent 1AC, Na_2_CO_3_ (492 mg, 4.64 mmol), and water (20 mL) were added to the reaction, and the temperature was increased to 120 °C. After completion of the reaction, the reaction mixture was cooled down to room temperature. The solvent was evaporated under a vacuum, and the residue was extracted by water and dichloromethane. The combined organic phases were dried with anhydrous Na_2_SO_4_ and evaporated in a vacuum. Further purification was carried out by column chromatography (SiO_2_, 3% MeOH in DCM) to provide the final product as a yellow solid (930 mg, 57%). Mp: above 300 °C. ^1^H NMR (400 MHz, DMSO, 25 °C) δ = 9.85 (t, *J* = 9.8 Hz, 1H), 8.91 (d, *J* = 6.6 Hz, 2H), 8.85 (d, *J* = 7.2 Hz, 1H), 8.61 (s, 1H), 8.18 (t, *J* = 6.4 Hz, 2H), 8.00 (d, *J* = 7.3 Hz, 2H), 7.92–7.85 (m, 1H), 7.62–7.53 (m, 1H), 4.64 (t, *J* = 6.9 Hz, 2H), 3.47 (t, *J* = 6.9 Hz, 2H). ^13^C NMR (101 MHz, DMSO, 25 °C) δ = 34.37, 111.17, 115.45, 119.76, 122.76, 126.11, 127.04, 128.07, 128.55, 128.76, 130.86, 132.38, 132.62, 133.06, 133.30, 135.78, 141.69, 144.04, 161.17, 163.30, 165.10. HRMS (ESI): *m*/*z* calcd for C_46_H_30_N_4_O_4_: 703.23266; found: 703.23270 [M+H]^+^.

#### 4.2.5. Synthesis of 1DiAC·Cl

**1DiAC** (1 g, 1.42 mmol) and iodomethane (403 mg, 2.84 mmol) were dissolved in anhydrous N, N-Dimethylformamide (30 mL). The reaction mixture was stirred at 90 °C for 24 h. The solvent was removed in a vacuum, and the pure product was obtained by washing it with dichloromethane, acetonitrile, and methanol. The filter cake was dissolved in methanol, and chloride salt was obtained via Amberlite IRA(400)Cl resin. The combined organic solution was evaporated and dried in a vacuum to give the orange solid (742 mg, 65%). Mp: above 300 °C. *Counterion exchange*: the solid was taken up in hot water, and a saturated solution of NH_4_PF_6_ in water was added dropwise until no further precipitate formed. The orange precipitate was then filtered and washed with water and diethyl ether. The filter cake was dried in a vacuum to give **1DiAC**·PF_6_. ^1^H NMR (400 MHz, CD_3_CN, 25 °C) δ = 9.89 (d, *J* = 9.1 Hz, 1H), 8.83 (d, *J* = 7.2 Hz, 1H), 8.51 (d, *J* = 5.1 Hz, 3H), 8.02 (d, *J* = 6.5 Hz, 2H), 7.91 (d, *J* = 7.2 Hz, 1H), 7.86 (dd, *J* = 9.1, 6.7 Hz, 1H), 7.81 (d, *J* = 8.5 Hz, 1H), 7.56–7.50 (m, 1H), 4.65 (t, *J* = 7.2 Hz, 2H), 4.26 (s, 3H), 3.48 (t, *J* = 7.1 Hz, 2H). ^13^C NMR (101 MHz, CD_3_CN, 25 °C) δ = 34.84, 40.72, 48.69, 116.51, 123.77, 126.99, 127.71, 129.01, 129.43, 129.69, 129.81, 131.10, 132.90, 133.51, 133.76, 134.13, 136.57, 145.00, 145.55, 161.18, 164.35, 164.76, 166.14. HRMS (ESI): *m*/*z* calcd for C_48_H_36_N_4_O_4_: 366.13628; found: 366.13671 [M-2PF_6_]^2+^.

#### 4.2.6. Synthesis of 2An Dione

Compound **2An Dione** was synthesized following a similar procedure to that used for **1An Dione**, with a 54% yield. Mp: 265 ± 1 °C. ^1^H NMR (400 MHz, DMSO, 25 °C) δ = 9.21 (s, 1H), 9.09 (d, *J* = 1.8 Hz, 1H), 8.53 (d, *J* = 8.6 Hz, 1H), 8.37 (d, *J* = 9.1 Hz, 1H), 8.11 (d, *J* = 6.6 Hz, 1H), 7.93–7.87 (m, 2H). ^13^C NMR (101 MHz, DMSO, 25 °C) δ = 122.05, 122.18, 124.73, 125.20, 126.59, 128.03, 128.13, 128.30, 130.28, 130.55, 132.12, 132.31, 134.21, 145.37, 187.21, 187.83. HRMS (ESI): *m*/*z* calcd for C_16_H_7_BrO_2_: 310.97022; found: 310.96978 [M+H]^+^.

#### 4.2.7. Synthesis of 2AC

Compound **2AC** was synthesized following a similar procedure to that used for **1An Anhydride** and **1AC**, with a 64% yield. Mp: 210 ± 0.5 °C. ^1^H NMR (400 MHz, CDCl_3_, 25 °C) δ = 10.21 (s, 1H), 8.76 (s, 1H), 8.75-8.72 (m, 1H), 8.55 (dd, *J* = 4.5, 1.6 Hz, 2H), 8.33 (dd, *J* = 8.4, 0.8 Hz, 1H), 7.94 (d, *J* = 8.9 Hz, 1H), 7.78–7.73 (m, 1H), 7.69 (dd, *J* = 8.9, 1.9 Hz, 1H), 7.37 (dd, *J* = 4.5, 1.5 Hz, 2H), 4.53–4.43 (m, 2H), 3.19–3.03 (m, 2H). ^13^C NMR (101 MHz, CDCl_3_, 25 °C) δ = 33.77, 40.93, 114.28, 122.38, 124.74, 126.05, 127.57, 128.83, 129.02, 129.07, 130.54, 130.75, 131.12, 133.95, 134.42, 135.51, 136.74, 148.82, 149.34, 163.42, 164.79. HRMS (ESI): *m*/*z* calcd for C_23_H_15_BrN_2_O_2_: 431.03897; found: 431.03895 [M+H]^+^.

#### 4.2.8. Synthesis of 2DiAC·Cl

Compound **2DiAC**·Cl was synthesized following a similar procedure to that used for **1DiAC** and **1DiAC·Cl**, with a 48% yield. Mp: above 300 °C. Compound **2DiAC**·PF_6_ was obtained following a similar procedure as for **1DiAC**· PF_6_. ^1^H NMR (400 MHz, CD_3_CN, 25 °C) δ = 10.17 (s, 1H), 8.85 (s, 1H), 8.48 (d, *J* = 7.0 Hz, 1H), 8.44 (d, *J* = 6.5 Hz, 2H), 8.34 (d, *J* = 8.1 Hz, 1H), 8.25 (d, *J* = 8.8 Hz, 1H), 8.05 (dd, *J* = 8.8, 1.5 Hz, 1H), 7.96 (d, *J* = 6.6 Hz, 2H), 7.71–7.63 (m, 1H), 4.61 (t, *J* = 7.1 Hz, 2H), 4.22–4.13 (m, 3H), 3.41 (t, *J* = 7.0 Hz, 2H). ^13^C NMR (101 MHz, CD_3_CN, 25 °C) δ = 34.75, 40.50, 48.58, 115.55, 120.92, 122.68, 125.60, 126.68, 126.78, 129.36, 130.00, 132.01, 132.55, 134.05, 134.59, 136.60, 137.71, 143.33, 145.43, 161.07, 164.14, 165.93. HRMS (ESI): *m*/*z* calcd for C_48_H_36_N_4_O_4_: 366.13628; found: 366.13611 [M-2PF_6_]^2+^.

#### 4.2.9. Synthesis of 9DiAn Dione

Compound **9DiAn Dione** was synthesized following a similar procedure to that used for **1An Dione**, with a 40% yield. Mp: above 300 °C. ^1^H NMR (400 MHz, CDCl_3_, 25 °C) δ = 9.42 (d, *J* = 8.6 Hz, 1H), 8.14 (d, *J* = 6.5 Hz, 1H), 7.94–7.86 (m, 1H), 7.57 (dd, *J* = 8.8, 6.7 Hz, 1H), 7.49 (ddd, *J* = 7.9, 6.3, 3.0 Hz, 1H), 7.36 (dd, *J* = 8.8, 5.5 Hz, 2H). ^13^C NMR (101 MHz, CDCl_3_, 25 °C) δ = 122.32, 124.53, 125.50, 127.17, 127.63, 128.43, 128.54, 128.57, 128.92, 130.82, 130.85, 132.39, 139.59, 146.18, 187.73, 188.73. HRMS (ESI): *m*/*z* calcd for C_32_H_14_O_4_: 463.09649; found: 463.09620 [M+H]^+^.

#### 4.2.10. Synthesis of 9DiAn Anhydride

Compound **9DiAn Dione** was synthesized following a similar procedure to that used for **1An Anhydride**, with an 80% yield. Mp: above 300 °C. ^1^H NMR (400 MHz, CDCl_3_, 25 °C) δ = 9.98 (d, *J* = 9.2 Hz, 1H), 8.80 (dd, *J* = 6.9, 1.2 Hz, 1H), 7.94 (ddd, *J* = 9.2, 6.6, 1.2 Hz, 1H), 7.58–7.48 (m, 1H), 7.48–7.38 (m, 2H), 7.19 (d, *J* = 8.7 Hz, 1H). ^13^C NMR (101 MHz, CDCl_3_, 25 °C) δ = 113.60, 119.53, 127.03, 127.12, 127.73, 128.65, 128.98, 130.73, 132.39, 132.48, 133.83, 134.30, 136.04, 142.69, 160.06, 160.67. HRMS (ESI): *m*/*z* calcd for C_32_H_14_O_6_: 495.08631; found: 495.08599 [M+H]^+^.

#### 4.2.11. Synthesis of 9DiAC

Compound **9DiAC** was synthesized following a similar procedure to that used for **1AC**, with a 65% yield. Mp: above 300 °C. ^1^H NMR (400 MHz, CDCl_3_, 25 °C) δ = 10.19 (d, *J* = 9.2 Hz, 1H), 8.78 (d, *J* = 6.6 Hz, 1H), 8.65 (s, 2H), 7.86 (dd, *J* = 8.8, 6.7 Hz, 1H), 7.56 (s, 2H), 7.47 (t, *J* = 7.5 Hz, 1H), 7.37 (dd, *J* = 12.5, 6.0 Hz, 2H), 7.17 (d, *J* = 8.6 Hz, 1H), 4.72–4.62 (m, 2H), 3.35–3.23 (m, 2H). ^13^C NMR (101 MHz, CDCl_3_, 25 °C) δ = 34.13, 40.94, 116.70, 122.78, 125.46, 126.70, 127.30, 127.81, 127.88, 128.25, 129.11, 131.69, 132.51, 133.48, 133.61, 134.08, 142.10, 147.79, 150.97, 163.56, 165.10. HRMS (ESI): *m*/*z* calcd for C_46_H_30_N_4_O_4_: 352.12063; found: 352.12032 [M+2H]^2+^.

#### 4.2.12. Synthesis of 9DiAC·Cl

Compound **9DiAC**·Cl was synthesized following a similar procedure to that used for **1DiAC**·Cl, with a 60% yield. Mp: above 300 °C. Compound **9DiAC**·PF_6_ was obtained following a similar procedure as for **1DiAC**· PF_6_. ^1^H NMR (400 MHz, CD_3_CN, 25 °C) δ = 10.12 (d, *J* = 9.2 Hz, 1H), 8.70 (dd, *J* = 6.9, 1.3 Hz, 1H), 8.51 (d, *J* = 6.6 Hz, 2H), 8.03 (d, *J* = 6.7 Hz, 2H), 7.92–7.84 (m, 1H), 7.54–7.47 (m, 1H), 7.45–7.35 (m, 2H), 7.19 (d, *J* = 8.4 Hz, 1H), 4.68 (t, *J* = 7.1 Hz, 2H), 4.25 (s, 3H), 3.49 (t, *J* = 7.1 Hz, 2H). ^13^C NMR (101 MHz, CD_3_CN, 25 °C) δ = 34.81, 40.77, 48.66, 117.74, 123.77, 127.62, 127.76, 128.47, 128.50, 129.28, 129.44, 130.10, 132.25, 133.22, 134.10, 134.19, 134.46, 142.85, 145.52, 161.13, 164.32, 166.09. HRMS (ESI): *m*/*z* calcd for C_48_H_36_N_4_O_4_: 366.13628; found: 366.13618 [M-2PF_6_]^2+^.

## Data Availability

Not applicable.

## Supplementary Materials

The following supporting information can be downloaded at: https://www.mdpi.com/article/10.3390/molecules28073217/s1, Scheme S1: The synthetic routes of the **1DiAC**∙Cl; Scheme S2: The synthetic routes of the **2DiAC**∙Cl; Scheme S3: The synthetic routes of the **9DiAC**∙Cl; Figure S1: Absorption spectra of (a) **1DiAC**∙Cl, (b) **2DiAC**∙Cl, and (c) **9DiAC**∙Cl in various solvents (c = 1 × 10^−5^ M). (d) Photographs of 1 mM **1DiAC**∙Cl, **2DiAC**∙Cl, and **9DiAC**∙Cl in N, N-dimethylacetamide (DMA), N, N-dimethylformamide (DMF), 1-propanol (1-PrOH), ethanol (EtOH), dimethyl sulfoxide (DMSO), methanol (CH_3_OH), and water (H_2_O); Figure S2. Normalized emission spectra of (a) **1DiAC**∙Cl, (b) **2DiAC**∙Cl, and (c) **9DiAC**∙Cl in various solvents (c = 1 × 10^−5^ M); Figure S3. (a) Absorption and (b) emission spectra of **2AC** in N, N-dimethylacetamide (DMA), N, N-dimethylformamide (DMF), 1-propanol (1-PrOH), ethanol (EtOH), dimethyl sulfoxide (DMSO), methanol (CH_3_OH), and water (H_2_O). c = 1 × 10^−5^ M; Table S1. Photophysical Data for the Excitation Center λ_Abs_, Emission Center λ_Em_, Stokes Shift (Δ*ν*_St_), the Absolute Quantum Yield (Φ_F_), Fluorescent Lifetime (τ), and Molar Absorptivity (ε_max_) of 1DiAC·Cl, 2DiAC·Cl, and 9DiAC·Cl in Different Solvents; Table S2. Photophysical Data for the Excitation Center λ_Abs_, Emission Center λ_Em_, Stokes Shift (Δ*ν*_St_), and Molar Absorptivity (ε_max_) of 2AC in Different Solvents; Table S3. Dielectric constant (Ɛ), Refractive Index of the Solvent (n), and Orientation Polarizability (Δf) of solvents; Figure S4: Lippert–Mataga plots for (a) **1DiAC**∙Cl, (b) **2DiAC**∙Cl, and (c) **9DiAC**∙Cl; Table S4: The Physical Parameters of the Solvents (Kamlet–Taft and Catalán); Table S5: Estimated Coefficients (y_0_, a_α_, b_β_, c_π*_), their Standard Errors, and Regression Coefficients (R^2^) for the Multilinear Analysis of *ν*_Abs_, *ν*_Em_, Δ*ν*_St_, and Φ_F_ of Investigated Rotors in N, N-dimethylacetamide (DMA), N, N-dimethylformamide (DMF), 1-propanol (1-PrOH), ethanol (EtOH), dimethyl sulfoxide (DMSO), methanol (CH_3_OH), and water (H_2_O) as a Function of Kamlet–Taft Solvent Scales; Table S6: Estimated Coefficients (y_0_, a_SA_, b_SB_, c_SP_, d_SDP_), their Standard Errors, and Regression Coefficients (R^2^) for the Multilinear Analysis of *ν*_Abs_, *ν*_Em_, Δ*ν*_St_, and Φ_F_ of Investigated Rotors in N, N-dimethylacetamide (DMA), N, N-dimethylformamide (DMF), 1-propanol (1-PrOH), ethanol (EtOH), dimethyl sulfoxide (DMSO), methanol (CH_3_OH), and water (H_2_O) as a Function of Catalán Solvent Scales; Table S7: Percentage Contribution of the Solvatochromic Parameters (Catalán Equation) Using Absorption, Emission Frequencies, and Absolute Fluorescent Quantum Yield; Figure S5: Absorption spectra of (a) **1DiAC**∙Cl, (b) **2DiAC**∙Cl, and (c) **9DiAC**∙Cl binary mixtures of water and glycerol in different ratios. c = 1 × 10^−5^ M; Figure S6: Emission spectra of **1DiAC**·Cl in binary mixtures of water and glycerol in different ratios. c = 1 × 10^−5^ M; Table S8: Viscosity in Different Fractions of Glycerol in Water at 25 °C; Figure S7: Emission spectra of (a) **1DiAC**·Cl, (b) **2DiAC**·Cl, and (c) **9DiAC**·Cl in binary mixtures of water and glycerol (v:v = 2:8, c = 1 × 10^−5^ M) at 25 and 80 °C; Figure S8: Emission spectra of (a) **1DiAC**·Cl, (b) **2DiAC**·Cl, and (c) **9DiAC**·Cl in DMF (c = 1 × 10^−5^ M) at 20 and 60 °C; Figure S9: Mixed space filling and stick representations of the X-ray crystal structures of **9DiAC**·PF_6_ as viewed from the front and the side. Hydrogen atoms and counterions have been omitted for clarity; Table S9: Crystallographic Parameters for **9DiAC**·PF_6_; Figure S10: ^1^H NMR spectrum of **1An Dione** (400 MHz, DMSO-d6, 298 K); Figure S11: ^1^H-^1^H COSY NMR spectrum of **1An Dione** (400 MHz, DMSO-d6, 298 K); Figure S12: ^13^C NMR Spectrum of **1An Dione** (101 MHz, DMSO-d6, 298 K); Figure S13: ^1^H NMR spectrum of **1An Anhydride** (400 MHz, DMSO-d6, 298 K); Figure S14: ^13^C NMR spectrum of **1An Anhydride** (101 MHz, DMSO-d6, 298 K); Figure S15: ^1^H NMR spectrum of **1AC** (400 MHz, CDCl_3_, 298 K); Figure S16: ^13^C NMR spectrum of **1AC** (101 MHz, CDCl_3_, 298 K); Figure S17: ^1^H NMR spectrum of **1DiAC** (400 MHz, DMSO-d6, 298 K); Figure S18: ^13^C NMR spectrum of **1DiAC** (101 MHz, DMSO-d6, 298 K); Figure S19: ^1^H NMR spectrum of **1DiAC∙PF_6_** (400 MHz, CD_3_CN, 298 K); Figure S20: ^13^C NMR spectrum of **1DiAC∙PF_6_** (101 MHz, CD_3_CN, 298 K); Figure S21: ^1^H NMR spectrum of **2An Dione** (400 MHz, DMSO-d6, 298 K); Figure S22: ^13^C NMR spectrum of **2An Dione** (101 MHz, DMSO-d6, 298 K); Figure S23: ^1^H NMR spectrum of **2AC** (400 MHz, CDCl_3_, 298 K); Figure S24: ^13^C NMR spectrum of **2AC** (101 MHz, CDCl_3_, 298 K); Figure S25: ^1^H NMR spectrum of **2DiAC∙PF_6_** (400 MHz, CD_3_CN, 298 K); Figure S26: ^13^C NMR spectrum of **2DiAC∙PF_6_** (101 MHz, CD_3_CN, 298 K); Figure S27: ^1^H NMR spectrum of **9DiAn Dione** (400 MHz, CDCl_3_, 298 K); Figure S28: ^13^C NMR spectrum of **9DiAn Dione** (101 MHz, CDCl_3_, 298 K); Figure S29: ^1^H NMR spectrum of **9DiAn Anhydride** (400 MHz, CDCl_3_, 298 K); Figure S30: ^13^C NMR spectrum of **9DiAn Anhydride** (101 MHz, CDCl_3_, 298 K); Figure S31: ^1^H NMR spectrum of **9DiAC** (400 MHz, CDCl_3_, 298 K); Figure S32: ^13^C NMR spectrum of **9DiAC** (101 MHz, CDCl_3_, 298 K); Figure S33: ^1^H NMR spectrum of **9DiAC∙PF_6_** (400 MHz, CD_3_CN, 298 K); Figure S34: ^13^C NMR spectrum of **9DiAC∙PF_6_** (101 MHz, CD_3_CN, 298 K); Figure S35: High resolution mass spectra of **1An Dione**; **Figure S36**: High resolution mass spectra of **1An Anhydride**; Figure S37: High resolution mass spectra of **1AC**; Figure S38: High resolution mass spectra of **1DiAC**; Figure S39: High resolution mass spectra of **1DiAC∙PF_6_**; Figure S40: High resolution mass spectra of **2An Dione**; Figure S41: High resolution mass spectra of **2AC**; Figure S42: High resolution mass spectra of **2DiAC∙PF_6_**; Figure S43: High resolution mass spectra of **9DiAn Dione**; Figure S44: High resolution mass spectra of **9DiAn Anhydride**; Figure S45: High resolution mass spectra of **9DiAC**; Figure S46: High resolution mass spectra of **9DiAC∙PF_6_**. References [38,39,40,41] are cited in the Supplementary Materials.

## Sample Availability

Samples of the compounds are not available from the authors.
