# Supplementary material for: Fluorescent Molecular Rotors Based on Hinged Anthracene Carboxyimides
Source: Molecules. 2023 Apr 4;28(7):3217. doi: 10.3390/molecules28073217 (PMC10096540; doi:10.3390/molecules28073217)
Supplement: Supplementary file 1 [file molecules-28-03217-s001.zip › molecules-2257764-supplementary.pdf]

Supporting Information for :

Fluorescent Molecular Rotors Based on Hinged Anthracene Carboxyimides

Yanhai Ni,[1] Wangjian Fang,[1] and Mark A. Olson[2,*]

[1] *School of Pharmaceutical Science and Technology, Tianjin University, 92 Weijin Road, Nankai District, Tianjin, 300072, P. R. China.*

[2] *Department of Physical and Environmental Sciences, Texas A&M University Corpus Christi, Corpus Christi, TX. USA*

Table of Contents

1. Syntheses....................2

2. Photophysical Properties and Solvatochromism....................4

3. Multilinear Analysis Using Kamlet-Taft and Catalán Parameters....................9

4. Viscosity Response of Fluorescent Molecular Rotors....................11

5. Viscosity-Related Temperature Response of Fluorescent Molecular Rotors....................13

6. Single Crystal Data....................14

7. NMR Spectroscopic Characterization Data....................16

8. High Resolution Mass Spectrometry Data....................29

References....................35

1. Syntheses

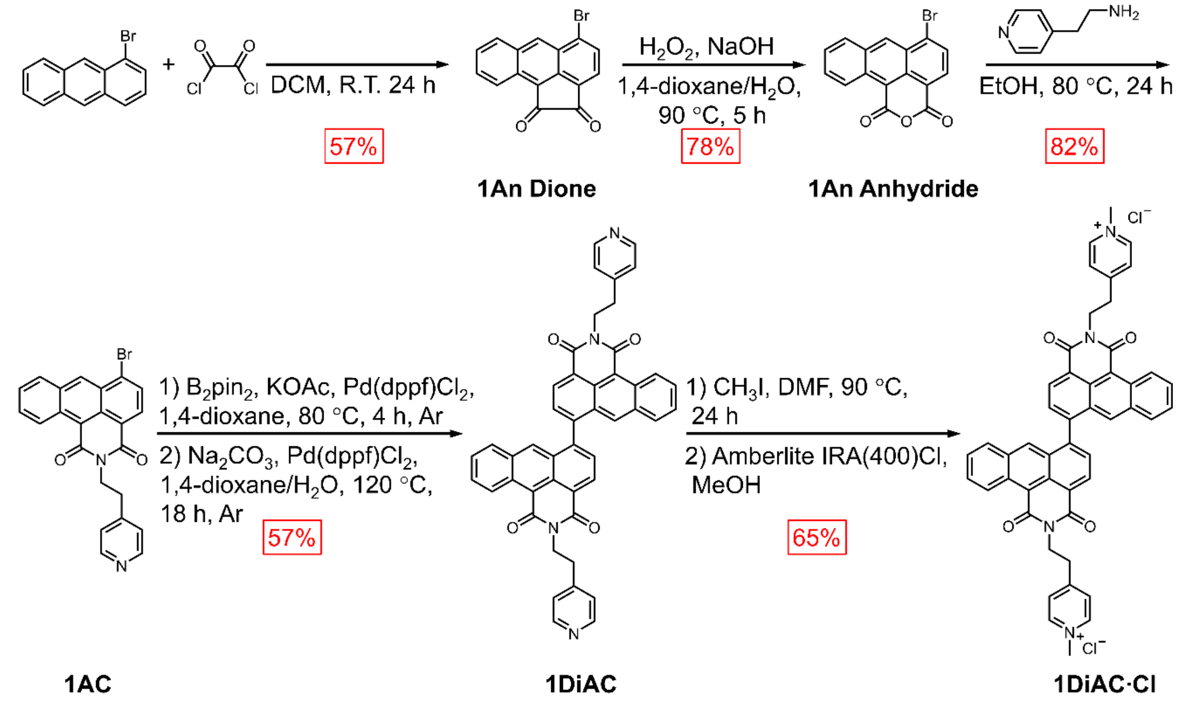

Scheme S1. The synthetic routes of the **1DiAC**·Cl.

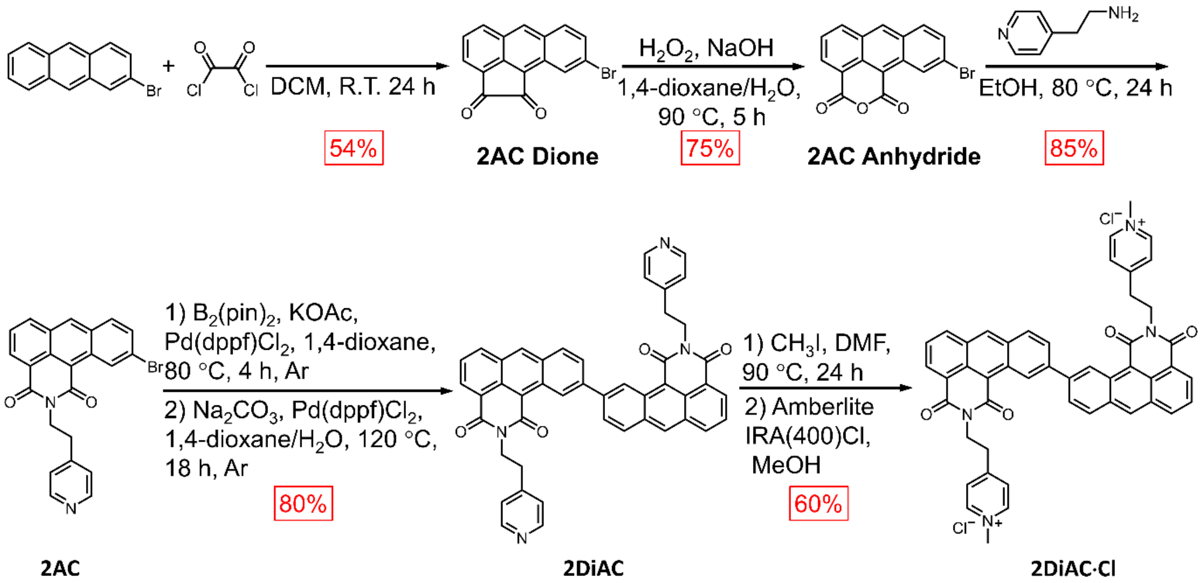

Scheme S2. The synthetic routes of the **2DiAC**·Cl.

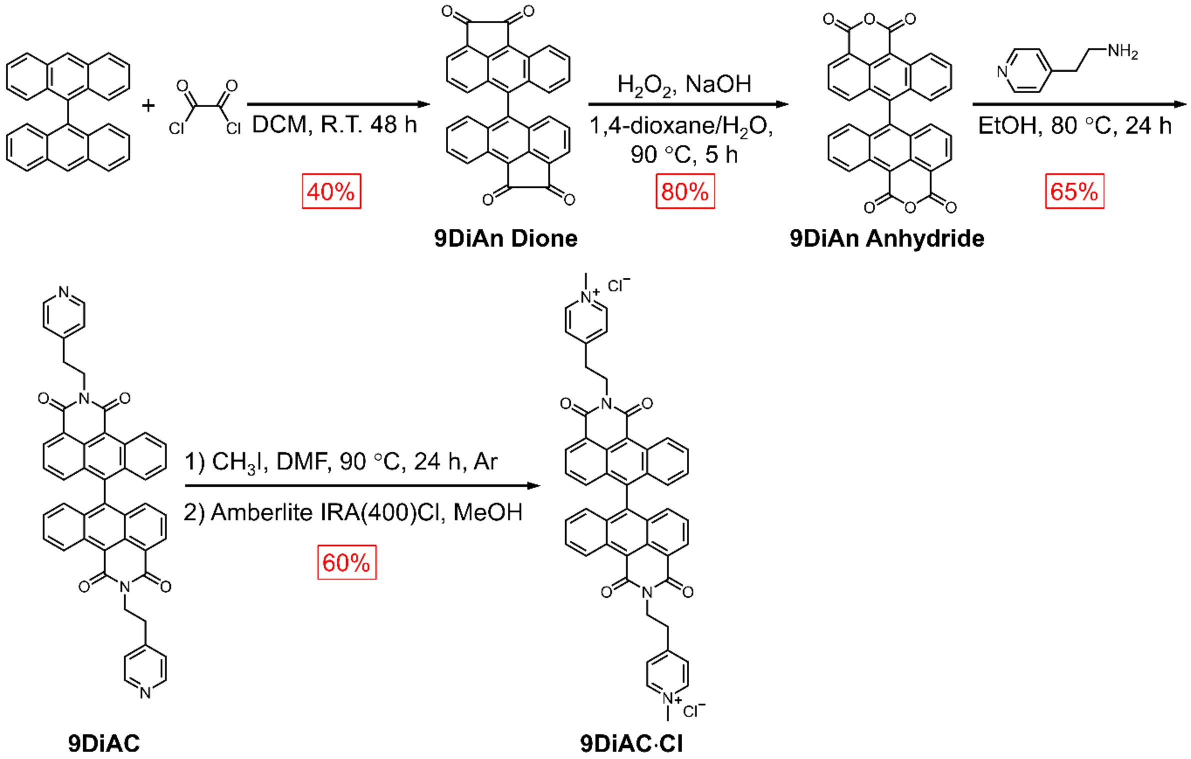

Scheme S3. The synthetic routes of the **9DiAC**·Cl.

2. Photophysical Properties and Solvatochromism

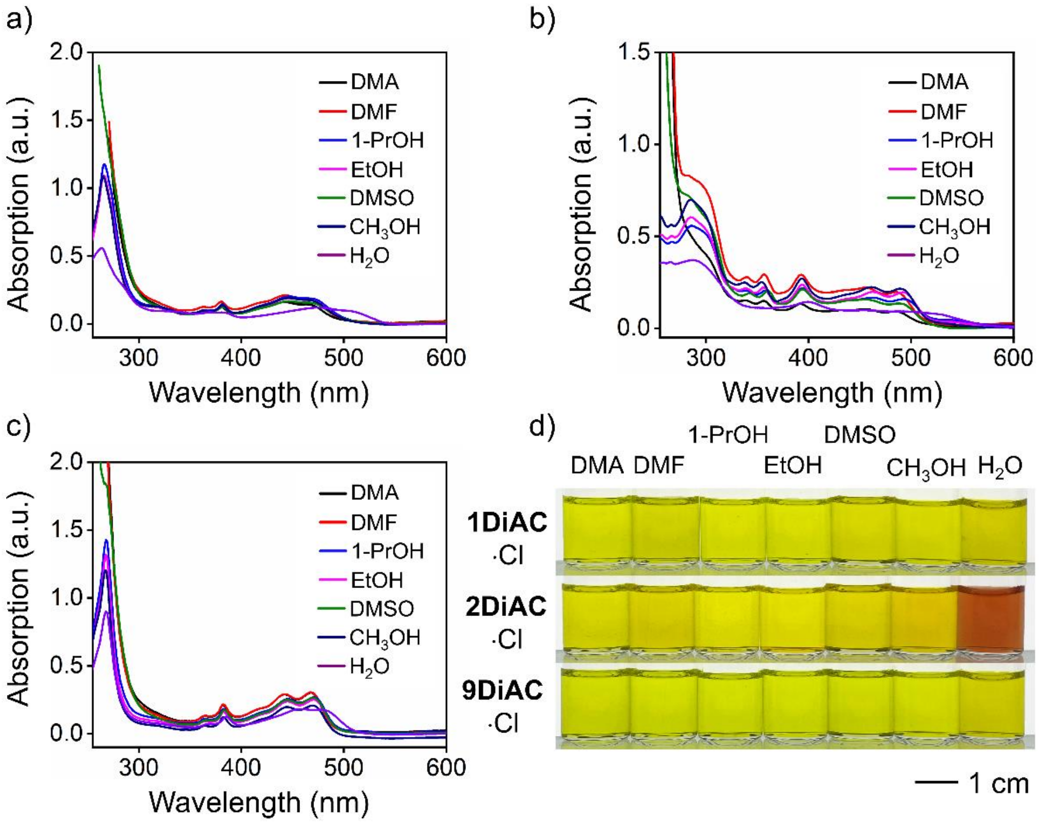

Figure S1. Absorption spectra of a) **1DiAC**·Cl, b) **2DiAC**·Cl, and c) **9DiAC**·Cl in various solvents (c = 1 × 10^{-5} M). d) Photographs of 1 mM **1DiAC**·Cl, **2DiAC**·Cl, and **9DiAC**·Cl in N, N-dimethylacetamide (DMA), N, N-dimethylformamide (DMF), 1-propanol (1-PrOH), ethanol (EtOH), dimethyl sulfoxide (DMSO), methanol (CH_3OH), and water (H_2O).

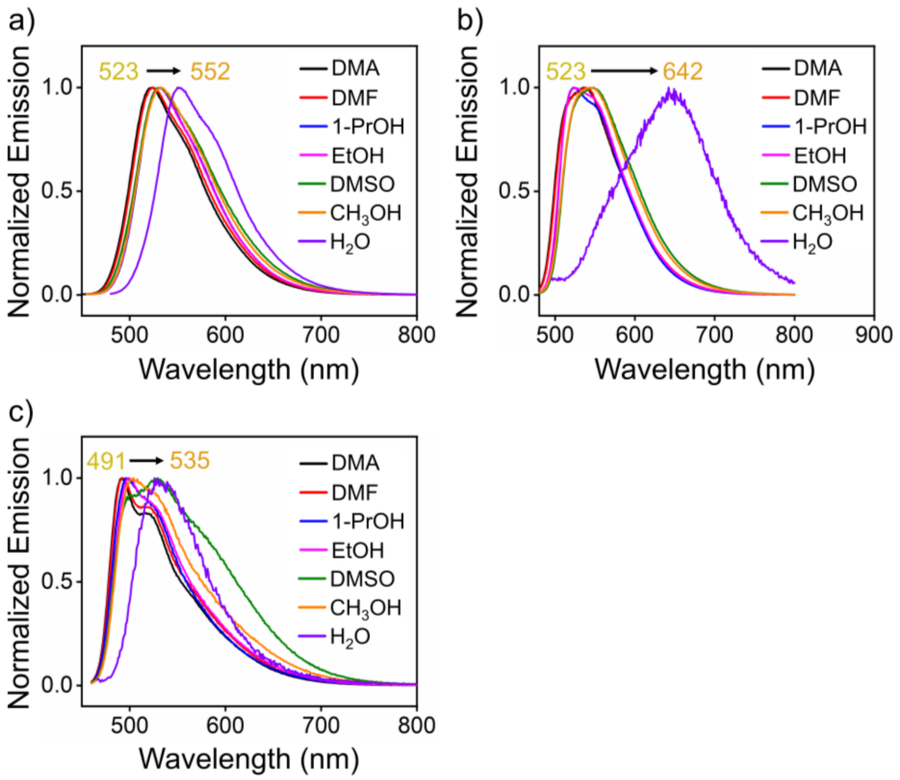

Figure S2. Normalized emission spectra of a) **1DiAC**·Cl, b) **2DiAC**·Cl, and c) **9DiAC**·Cl in various solvents (c = 1 × 10^{-5} M).

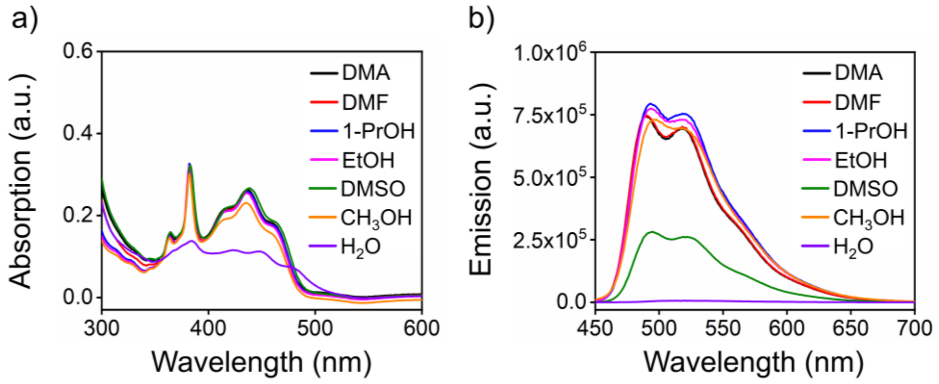

Figure S3. a) Absorption and b) emission spectra of **2AC** in N, N-dimethylacetamide (DMA), N, N-dimethylformamide (DMF), 1-propanol (1-PrOH), ethanol (EtOH), dimethyl sulfoxide (DMSO), methanol (CH_3OH), and water (H_2O). c = 1 × 10^{-5} M.

Table S1. Photophysical Data for the Excitation Center λ_{Abs}, Emission Center λ_{Em}, Stokes Shift ($\Delta\nu_{St}$), the Absolute Quantum Yield (Φ_F), Fluorescent Lifetime (τ), and Molar Absorptivity (ε_{max}) of 1DiAC·Cl, 2DiAC·Cl, and 9DiAC·Cl in Different Solvents.

Parameters	DMA	DMF	1-PrOH	EtOH	DMSO	CH_3OH	H_2O
λ_{Abs} of **1DiAC**·Cl / nm [a]	442	446	447	446	446	446	475
λ_{Em} of **1DiAC**·Cl / nm [a]	522	524	531	531	531	532	552
$\Delta\nu_{St}$/cm^{-1} [b]	3467	3338	3539	3589	3625	3625	2937
Φ_F of **1DiAC**·Cl [c]	0.6023	0.5187	0.4994	0.4512	0.2095	0.1396	0.1312
τ of **1DiAC**·Cl / ns	7.00	6.48	4.79	4.02	3.6	5.67	4.54
$\varepsilon_{max} \times 10^4$ of **1DiAC**·Cl / M^{-1} cm^{-1}	1.61	2.1	2.01	1.89	1.68	1.91	1.21
λ_{Abs} of **2DiAC**·Cl / nm [a]	456	456	464	463	456	461	489
λ_{Em} of **2DiAC**·Cl / nm [a]	536	538	523	525	547	543	642
$\Delta\nu_{St}$/cm^{-1} [b]	3273	3342	2431	2551	3648	3276	4874
Φ_F of **2DiAC**·Cl [c]	0.6801	0.6092	0.6032	0.5782	0.4103	0.2825	0.0081
τ of **2DiAC**·Cl / ns	7.28	6.02	5.32	5.87	5.38	5.01	5.19
$\varepsilon_{max} \times 10^4$ of **2DiAC**·Cl / M^{-1} cm^{-1}	1.04	2.21	1.67	1.98	1.56	2.24	0.92
λ_{Abs} of **9DiAC**·Cl / nm [a]	442	442	446	445	445	444	458
λ_{Em} of **9DiAC**·Cl / nm [a]	491	492	498	500	498	505	535
$\Delta\nu_{St}$/cm^{-1} [b]	2258	2299	2341	2472	2392	2721	3142
Φ_F of **9DiAC**·Cl [c]	0.2536	0.1643	0.1525	0.1234	0.0642	0.0018	0.0001
τ of **9DiAC**·Cl / ns	7.06	6.95	3.71	3.41	4.87	6.00	6.66
$\varepsilon_{max} \times 10^4$ of **1DiAC**·Cl / M^{-1} cm^{-1}	2.89	2.91	2.56	2.38	2.49	1.96	1.82

[a]All spectra in solution were measured using a 1 cm path length quartz cuvette at a concentration of 1 × 10^{-5} M at 298 K. [b] $\Delta\nu_{St} = \nu Abs_{max} - \nu Em_{max}$. [c] Φ = Absolute fluorescence quantum yield obtained using an integrating sphere.

Table S2. Photophysical Data for the Excitation Center λ_{Abs}, Emission Center λ_{Em}, Stokes Shift ($\Delta\nu_{St}$), and Molar Absorptivity (ε_{max}) of 2AC in Different Solvents.

Parameters	DMA	DMF	1-PrOH	EtOH	DMSO	CH_3OH	H_2O
λ_{Abs} of **2AC** / nm ^{a}	382	381	382	382	383	382	384
λ_{Em} of **2AC** / nm ^{a}	490	490	493	494	495	496	522
$\Delta\nu_{St}$/cm$^{-1\,b}$	5770	5839	5894	5935	5907	6016	6885
$\varepsilon_{max} \times 10^4$ of **2AC** / M^{-1} cm^{-1}	1.05	1.03	1.09	1.07	1.08	1.00	1.06

^{a}All spectra in solution were measured using a 1 cm path length quartz cuvette at a concentration of 1×10^{-5} M at 298 K. ^{b} $\Delta\nu_{St} = \nu Abs_{max} - \nu Em_{max}$.

Table S3. Dielectric constant (Ɛ), Refractive Index of the Solvent (n), and Orientation Polarizability (Δf) of solvents.

Parameters	DMA	DMF	1-PrOH	EtOH	DMSO	CH_3OH	H_2O
Ɛ	38.3	36.7	20.45	24.55	46.68	32.6	78.36
n	1.438	1.43	1.384	1.359	1.479	1.327	1.34
Δf	0.794	0.793	0.749	0.777	0.797	0.812	0.858

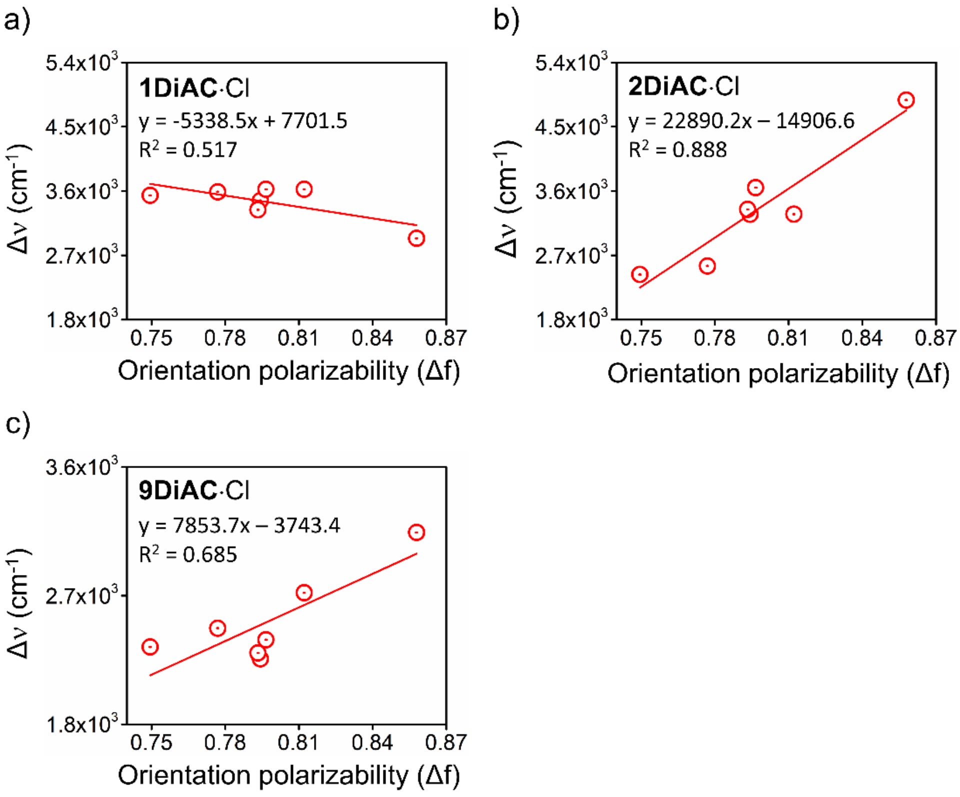

Figure S4. Lippert-Mataga plots for a) **1DiAC**·Cl, b) **2DiAC**·Cl, and c) **9DiAC**·Cl.

3. Multilinear Analysis Using Kamlet-Taft and Catalán Parameters

Table S4. The Physical Parameters of the Solvents (Kamlet-Taft and Catalán).

Solvent	Kamlet-Taft Parameter			Catalan Parameter			
	α	β	π^*	SA	SB	SP	SDP
N, N-dimethylacetamide	0	0.76	0.88	0.028	0.65	0.763	0.987
N, N-dimethylformamide	0	0.69	0.88	0.031	0.613	0.759	0.977
1-propanol	0.76	0.84	0.48	0.367	0.782	0.658	0.748
ethanol	0.86	0.75	0.54	0.4	0.658	0.633	0.783
dimethyl sulfoxide	0	0.76	1	0.072	0.647	0.83	1
methanol	0.98	0.66	0.6	0.605	0.545	0.608	0.904
water	1.17	0.47	1.09	1.062	0.025	0.681	0.997

Table S5. Estimated Coefficients (y_0, a_α, b_β, c_{π^*}), Their Standard Errors, and Regression Coefficients (R^2) for the multilinear analysis of ν_{Abs}, ν_{Em}, $\Delta\nu_{St}$, and Φ_F of Investigated Rotors in N, N-dimethylacetamide (DMA), N, N-dimethylformamide (DMF), 1-propanol (1-PrOH), ethanol (EtOH), dimethyl sulfoxide (DMSO), methanol (CH_3OH), and water (H_2O) as a Function of Kamlet-Taft Solvent Scalesb.

Parameters	y_0 / cm^{-1}	a_α	b_β	c_{π^*}	R^2
ν_{Abs} of **1DiAC**·Cl	24890 ± 2270	-1008 ± 390	-689 ± 1994	-2066 ± 919	0.839
ν_{Em} of **1DiAC**·Cl	20915 ± 898	-765 ± 154	-866 ± 788	-1376 ± 363	0.936
$\Delta\nu_{St}$ of **1DiAC**·Cl	3975 ± 2109	-243 ± 363	177 ± 1852	-689 ± 854	0.291
Φ_F of **1DiAC**·Cl	1.11 ± 2.03	-0.30 ± 0.35	-0.22 ± 1.78	-0.54 ± 0.82	0.53
ν_{Abs} of **2DiAC**·Cl	24609 ± 2330	-1196 ± 400	-1285 ± 2046	-1890 ± 944	0.825
ν_{Em} of **2DiAC**·Cl	22611 ± 2171	-1685 ± 373	-319 ± 1906	-4718 ± 879	0.972
$\Delta\nu_{St}$ of **2DiAC**·Cl	1999 ± 1608	489 ± 276	-1603 ± 1412	2827 ± 651	0.965
Φ_F of **2DiAC**·Cl	1.18 ± 1.23	-0.39 ± 0.21	0.06 ± 1.08	-0.72 ± 0.50	0.88
ν_{Abs} of **9DiAC**·Cl	24989 ± 951	-731 ± 163	-1489 ± 835	-1436 ± 385	0.891
ν_{Em} of **9DiAC**·Cl	22741 ± 617	-1221 ± 106	-838 ± 542	-2031 ± 250	0.990
$\Delta\nu_{St}$ of **9DiAC**·Cl	2248 ± 571	490 ± 98	-651 ± 502	594 ± 231	0.971
Φ_F of **9DiAC**·Cl	0.25 ± 0.91	-0.13 ± 0.16	0.06 ± 0.80	-0.14 ± 0.37	0.57

Table S6. Estimated Coefficients (y_0, a_{SA}, b_{SB}, c_{SP}, d_{SDP}), Their Standard Errors, and Regression Coefficients (R^2) for the multilinear analysis of ν_{Abs}, ν_{Em}, $\Delta\nu_{St}$, and Φ_F of Investigated Rotors in N, N-dimethylacetamide (DMA), N, N-dimethylformamide (DMF), 1-propanol (1-PrOH), ethanol (EtOH), dimethyl sulfoxide (DMSO), methanol (CH_3OH), and water (H_2O) as a Function of Catalán Solvent Scales.

Parameters	y_0 / cm^{-1}	a_{SA}	b_{SB}	c_{SP}	d_{SDP}	R^2
ν_{Abs} of **1DiAC**·Cl	20904 ± 1739	-395 ± 519	2304 ± 790	-3453 ± 1199	2879 ± 1057	0.962
ν_{Em} of **1DiAC**·Cl	20892 ± 1155	-1196 ± 345	-77 ± 525	-2554 ± 796	227 ± 701	0.958
$\Delta\nu_{St}$ of **1DiAC**·Cl	13 ± 2053	801 ± 613	2380 ± 933	-898 ± 1416	2652 ± 1248	0.733
Φ_F of **1DiAC**·Cl	5.05 ± 2.45	-1.54 ± 0.73	-1.84 ± 1.12	-1.25 ± 1.69	-2.42 ± 1.49	0.82
ν_{Abs} of **2DiAC**·Cl	19177 ± 1849	-405 ± 552	2208 ± 840	-2602 ± 1274	3449 ± 1123	0.956
ν_{Em} of **2DiAC**·Cl	21582 ± 2089	-1662 ± 624	3051 ± 950	-6244 ± 1440	-45 ± 1269	0.990
$\Delta\nu_{St}$ of **2DiAC**·Cl	-2405 ± 875	1257 ± 261	-843 ± 397	3642 ± 603	3494 ± 531	0.996
Φ_F of **2DiAC**·Cl	3.68 ± 1.36	-1.21 ± 0.41	-0.93 ± 0.62	-1.11 ± 0.94	-1.62 ± 0.83	0.96
ν_{Abs} of **9DiAC**·Cl	22734 ± 434	-608 ± 130	673 ± 197	-2727 ± 299	1595 ± 264	0.991
ν_{Em} of **9DiAC**·Cl	21483 ± 566	-1479 ± 169	-583 ± 257	-2782 ± 390	656 ± 344	0.997
$\Delta\nu_{St}$ of **9DiAC**·Cl	1251 ± 982	871 ± 293	90 ± 3446	55 ± 677	939 ± 597	0.976
Φ_F of **9DiAC**·Cl	1.77 ± 1.39	-0.61 ± 0.41	-0.68 ± 0.63	-0.41 ± 0.96	-0.84 ± 0.84	0.74

Table S7. Percentage Contribution of the Solvatochromic Parameters (Catalán Equation) Using Absorption, Emission Frequencies, and Absolute Fluorescent Quantum Yield.

Parameters	P_{SA} / %	P_{SB} / %	P_{SP} / %	P_{SDP} / %
ν_{Abs} of **1DiAC**·Cl	4.37	25.51	38.23	31.88
ν_{Em} of **1DiAC**·Cl	29.50	1.90	63.00	5.60
Φ_F of **1DiAC**·Cl	21.84	26.10	17.73	34.33
ν_{Abs} of **2DiAC**·Cl	4.67	25.48	30.03	39.81
ν_{Em} of **2DiAC**·Cl	15.11	27.73	56.75	0.41
Φ_F of **2DiAC**·Cl	24.85	19.09	22.79	33.26
ν_{Abs} of **9DiAC**·Cl	10.85	12.01	48.67	28.47
ν_{Em} of **9DiAC**·Cl	26.89	10.60	50.58	11.93
Φ_F of **9DiAC**·Cl	24.02	26.77	16.14	33.07

4. Viscosity Response of Fluorescent Molecular Rotors

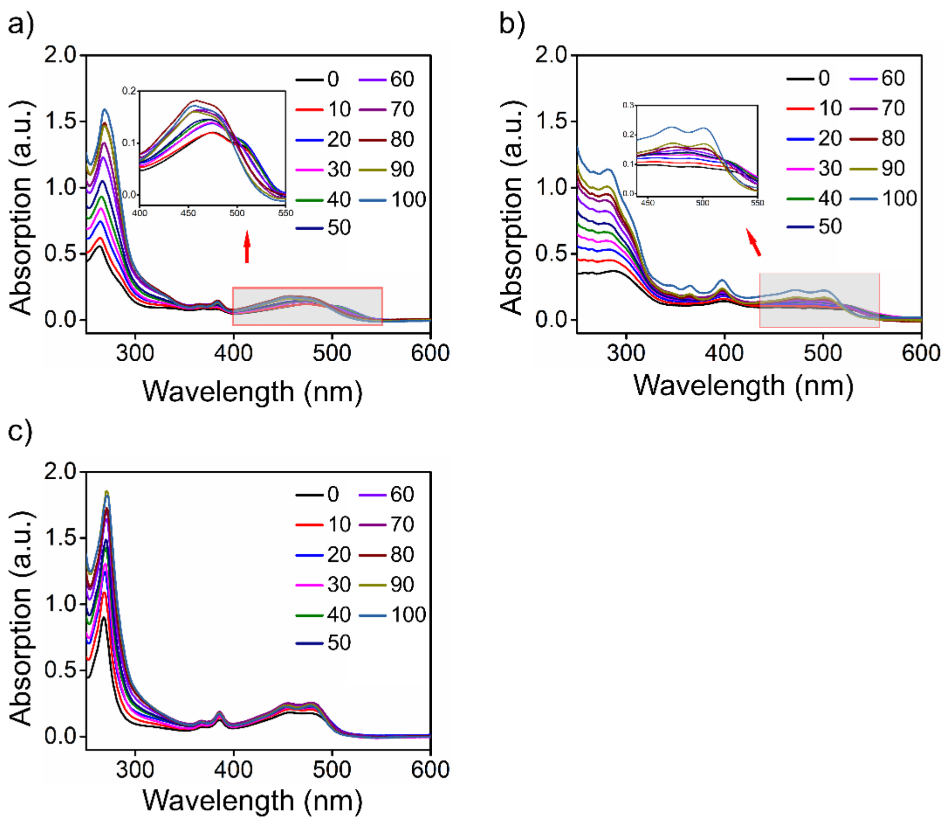

Figure S5. Absorption spectra of a) **1DiAC**·Cl, b) **2DiAC**·Cl, and c) **9DiAC**·Cl binary mixtures of water and glycerol in different ratios. c = 1 × 10^{-5} M.

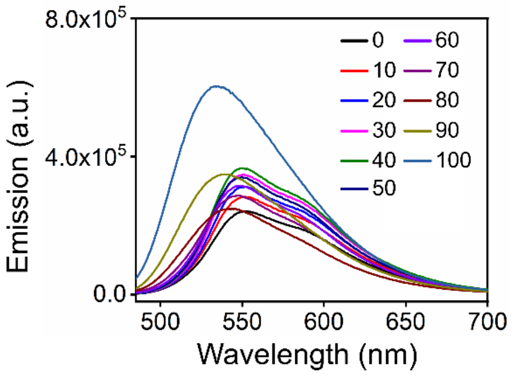

Figure S6. Emission spectra of **1DiAC**·Cl in binary mixtures of water and glycerol in different ratios. c = 1 × 10^{-5} M.

Table S8. Viscosity in Different Fractions of Glycerol in Water at 25 °C.

Volume Fraction /%	Viscosity / mPa·s
0	0.89
10	1.17
20	1.58
30	2.16
40	3.25
50	5.04
60	10.6
70	17.95
80	46.7
90	168.6
100	956.1

5. Viscosity-Related Temperature Response of Fluorescent Molecular Rotors

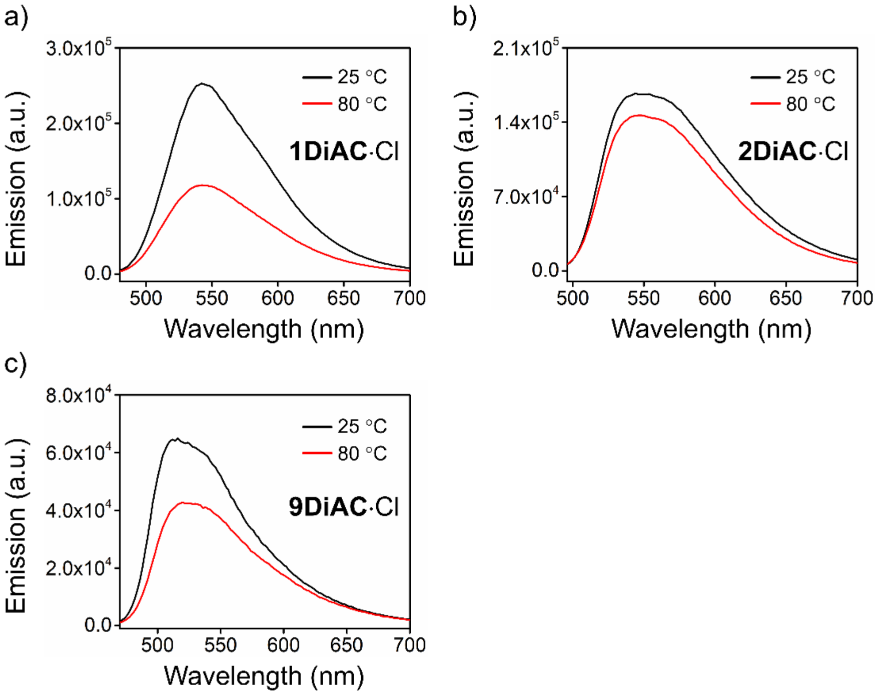

Figure S7. Emission spectra of a) **1DiAC**·Cl, b) **2DiAC**·Cl, and c) **9DiAC**·Cl in binary mixtures of water and glycerol (v : v = 2 : 8, c = 1 × 10^{-5} M) at 25 and 80 °C.

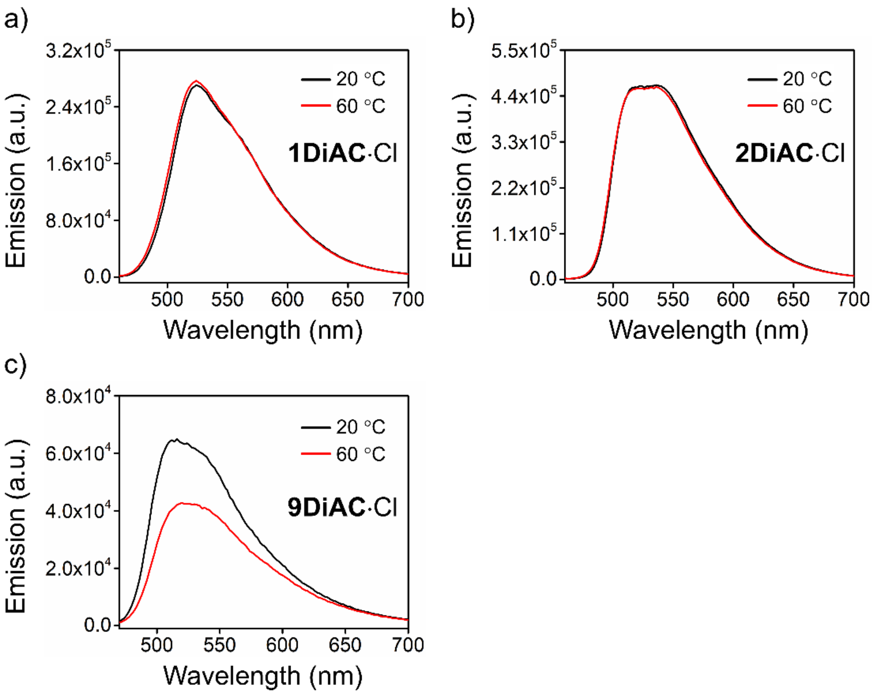

Figure S8. Emission spectra of a) **1DiAC**·Cl, b) **2DiAC**·Cl, and c) **9DiAC**·Cl in DMF (c = 1 × 10^{-5} M) at 20 and 60 °C.

6. Single Crystal Data

Single crystals suitable for X-ray diffraction were selected and X-ray diffraction intensity data was collected on a Rigaku XtaLAB FRX diffractometer equipped with a Hypix6000HE detectorand Rigaku MM-007 rotating anode diffractometer equipped with a Rigaku Pilatus 200K hybridphoton counting detector, using Cu Kα radiation (λ = 1.54184 Å). **9DiAC**·PF_6 crystals were grown from slow evaporation from dichloromethane and acetonitrile. Using Olex2,[1] structures were solved either with the ShelXT[2] by direct methods. The hydrogen atoms were set in calculated positions and refinedas riding atoms with a common fixed isotropic thermal parameter. Moredetails of the datacollection and structural refinement of all molecules can be found in Tables S7.

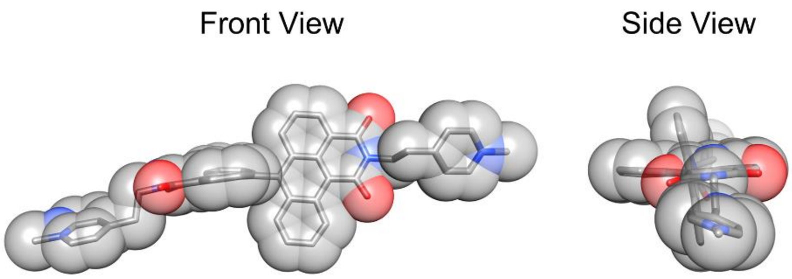

Figure S9. Mixed space filling and stick representations of the X-ray crystal structures of **9DiAC**·PF_6 as viewed from the front and the side. Hydrogen atoms and counterions have been omitted for clarity.

Table S9. Crystallographic Parameters for 9DiAC·PF_6.

Empirical formula	$C_{30}H_{26}N_4O_4$
Formula weight	506.55
Temperature/K	293(2)
Crystal system	monoclinic
Space group	$P2_1/n$
a/Å	12.6741(2)
b/Å	13.4071(2)
c/Å	16.1884 (3)
α/°	90
β/°	90.2360 (10)
γ/°	90
Volume/Å^3	2750.76(8)
Z	4
ρ_{calc}g/cm^3	1.223
μ/mm^{-1}	0.672
F(000)	1064.0
Crystal size/mm^3	0.3 × 0.2 × 0.2
Radiation	Cu Kα (λ = 1.54184)
2θ range for data collection/°	8.844 to 133.194
Index ranges	-14 ≤ h ≤ 15, -15 ≤ k ≤ 15, -19 ≤ l ≤ 16
Reflections collected	11657
Independent reflections	4616 [R_{int} = 0.0187, R_{sigma} = 0.0219]
Data/restraints/parameters	4616/0/343
Goodness-of-fit on F^2	1.054
Final R indexes [I>=2σ (I)]	R_1 = 0.0616, wR_2 = 0.1806
Final R indexes [all data]	R_1 = 0.0669, wR_2 = 0.1867
Largest diff. peak/hole / e Å^{-3}	0.23/-0.32
CCDC No.	2203337

7. NMR Spectroscopic Characterization Data

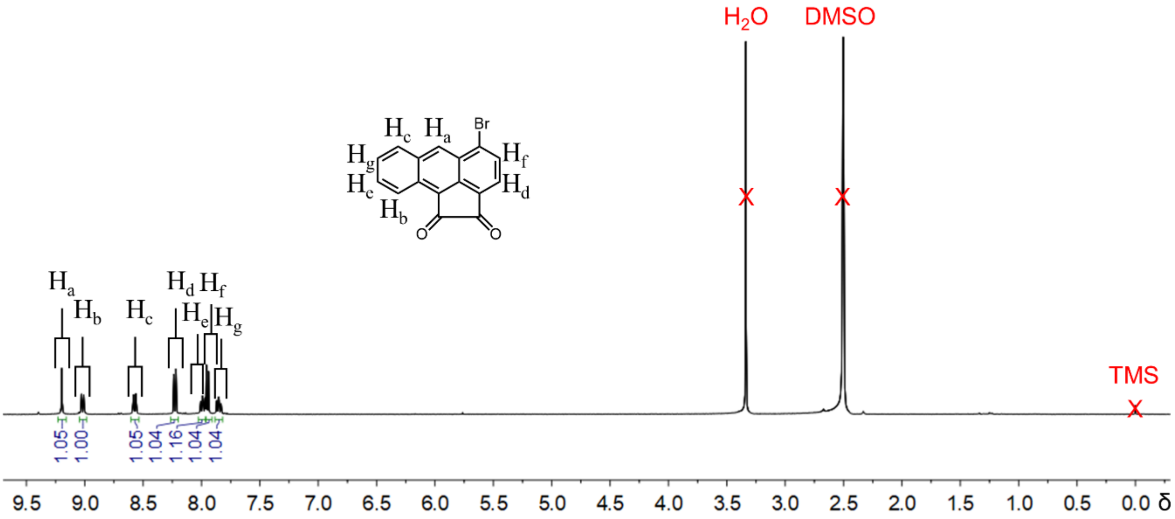

Figure S10. ^{1}H NMR spectrum of **1An Dione** (400 MHz, DMSO-d6, 298 K).

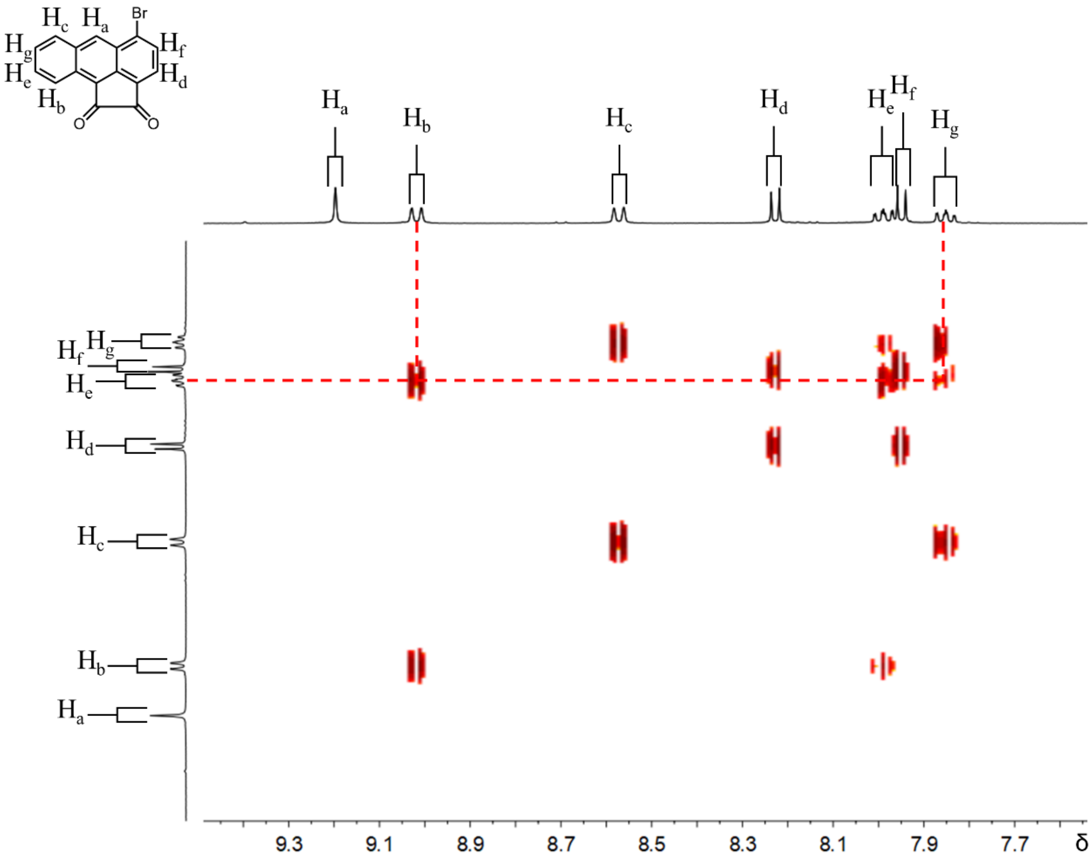

Figure S11. ^{1}H-^{1}H COSY NMR spectrum of **1An Dione** (400 MHz, DMSO-d6, 298 K).

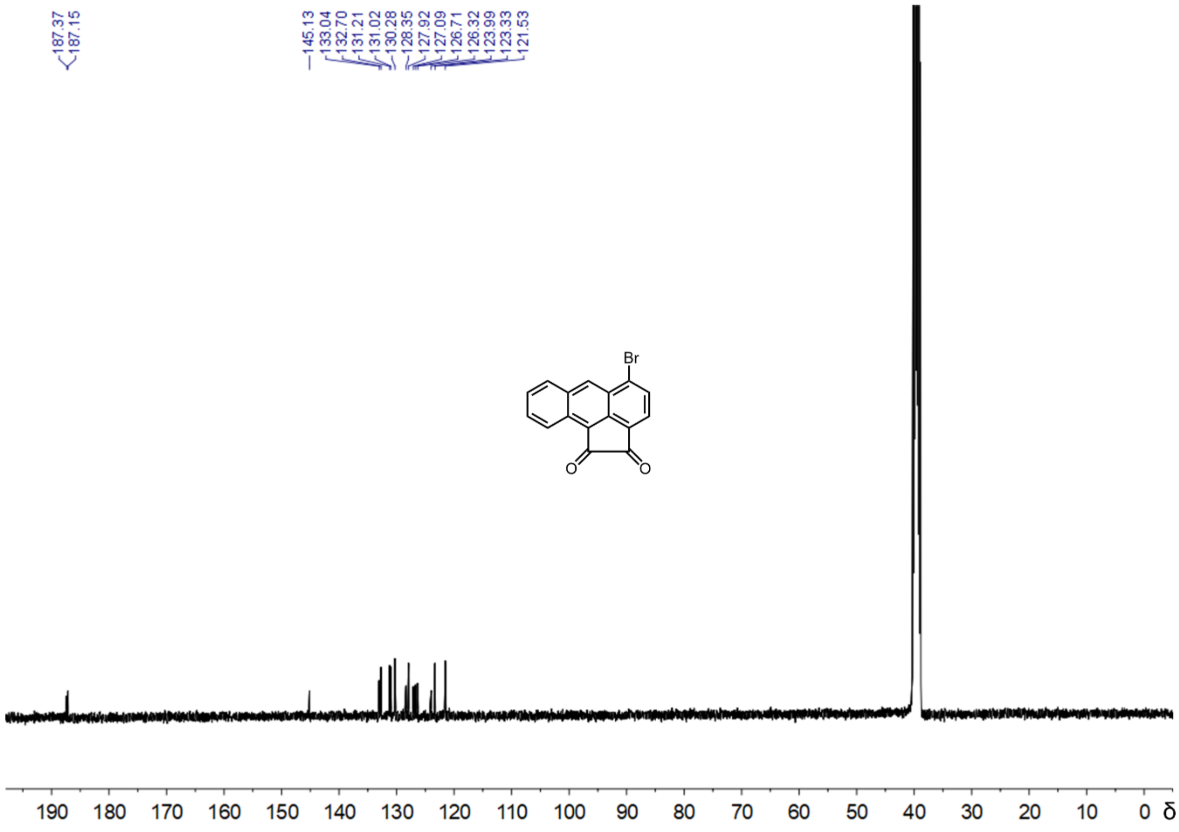

Figure S12. ^{13}C NMR Spectrum of **1An Dione** (101 MHz, DMSO-d6, 298 K).

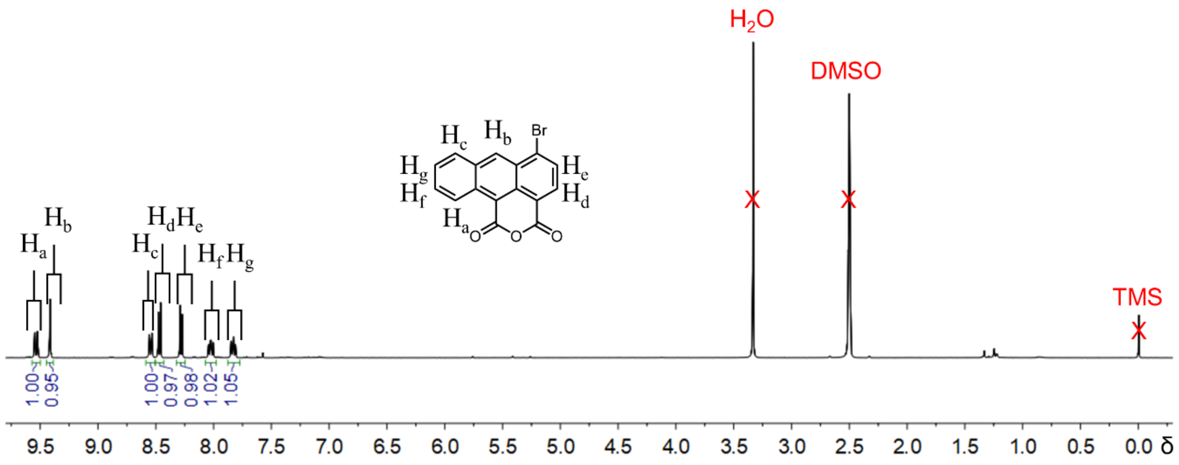

Figure S13. ^{1}H NMR spectrum of **1An Anhydride** (400 MHz, DMSO-d6, 298 K).

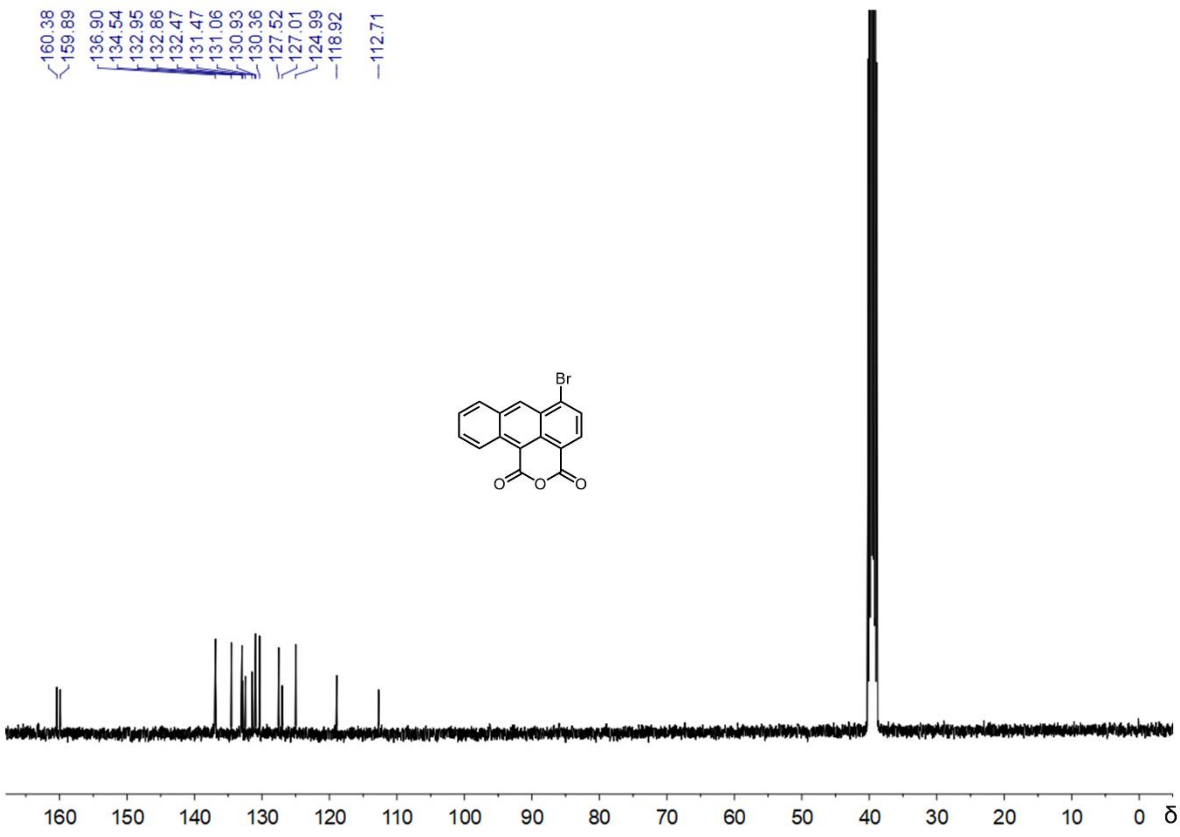

Figure S14. ^{13}C NMR Spectrum of **1An Anhydride** (101 MHz, DMSO-d6, 298 K).

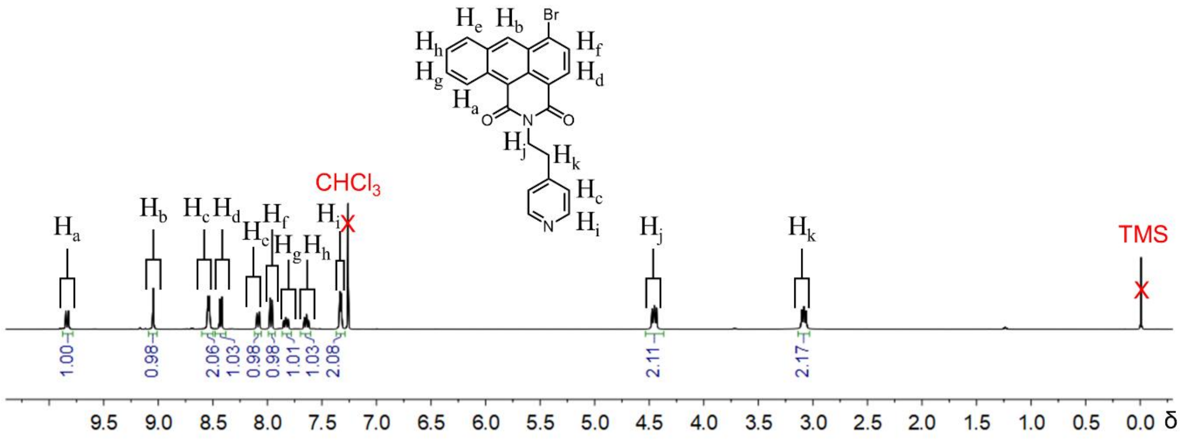

Figure S15. ^{1}H NMR spectrum of **1AC** (400 MHz, $CDCl_3$, 298 K).

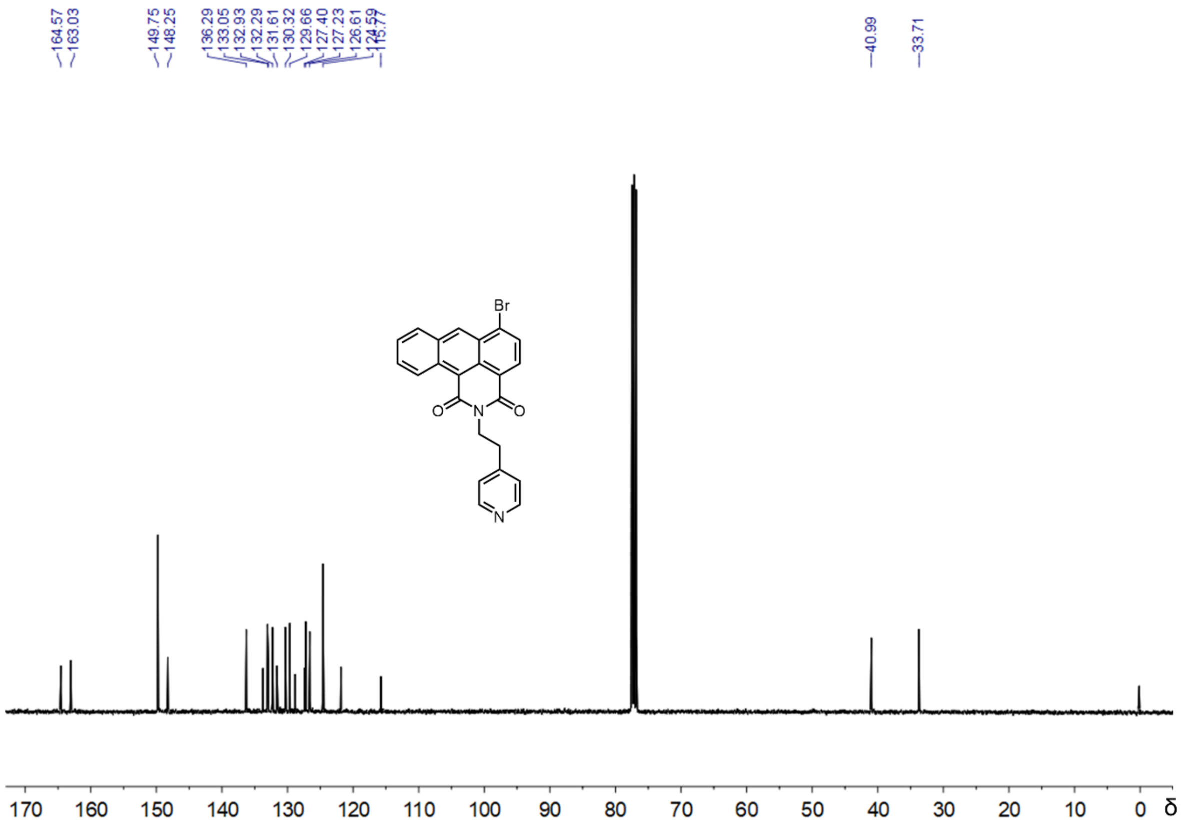

Figure S16. ^{13}C NMR Spectrum of **1AC** (101 MHz, $CDCl_3$, 298 K).

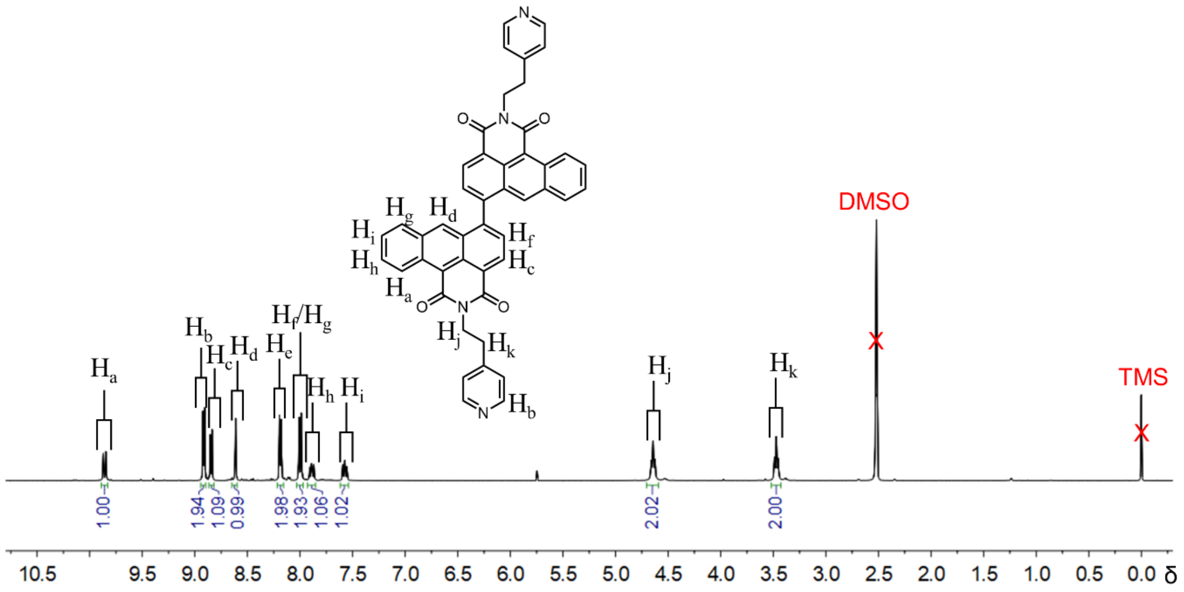

Figure S17. ^{1}H NMR spectrum of **1DiAC** (400 MHz, DMSO-d6, 298 K).

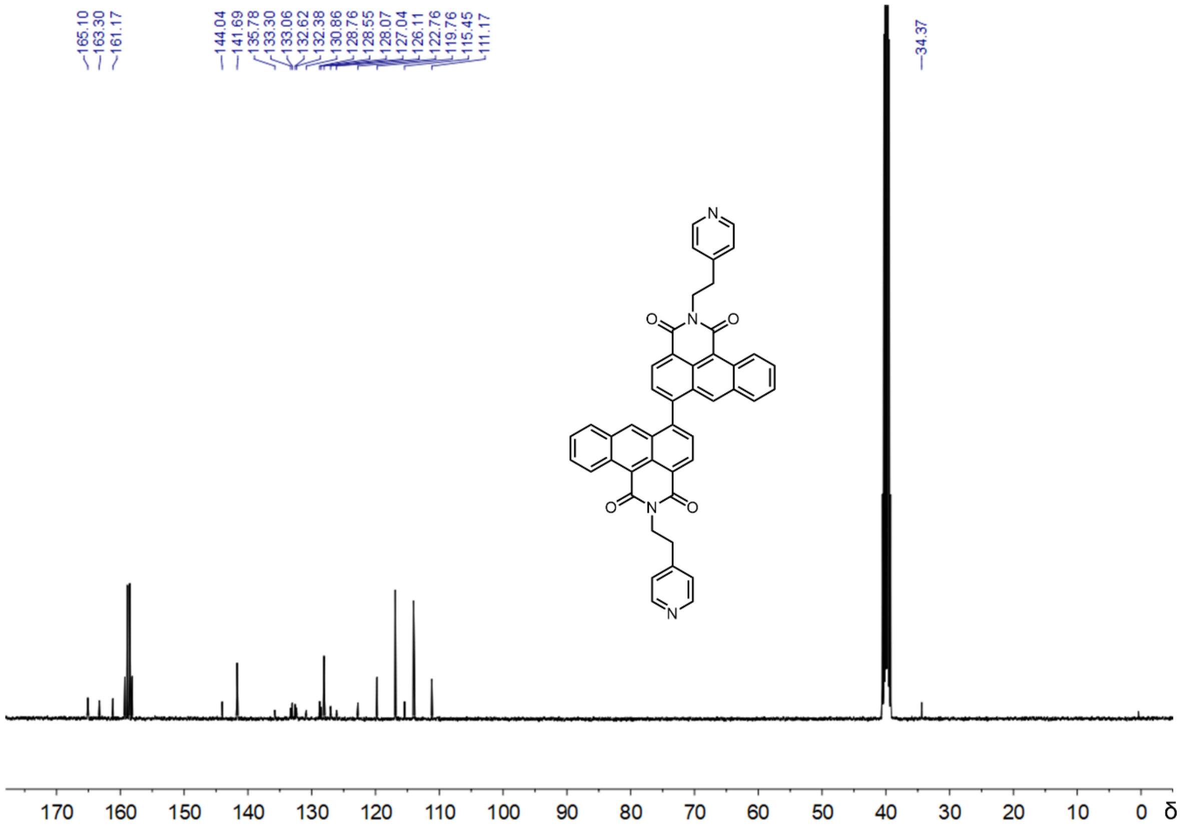

Figure S18. ^{13}C NMR Spectrum of **1DiAC** (101 MHz, DMSO-d6, 298 K).

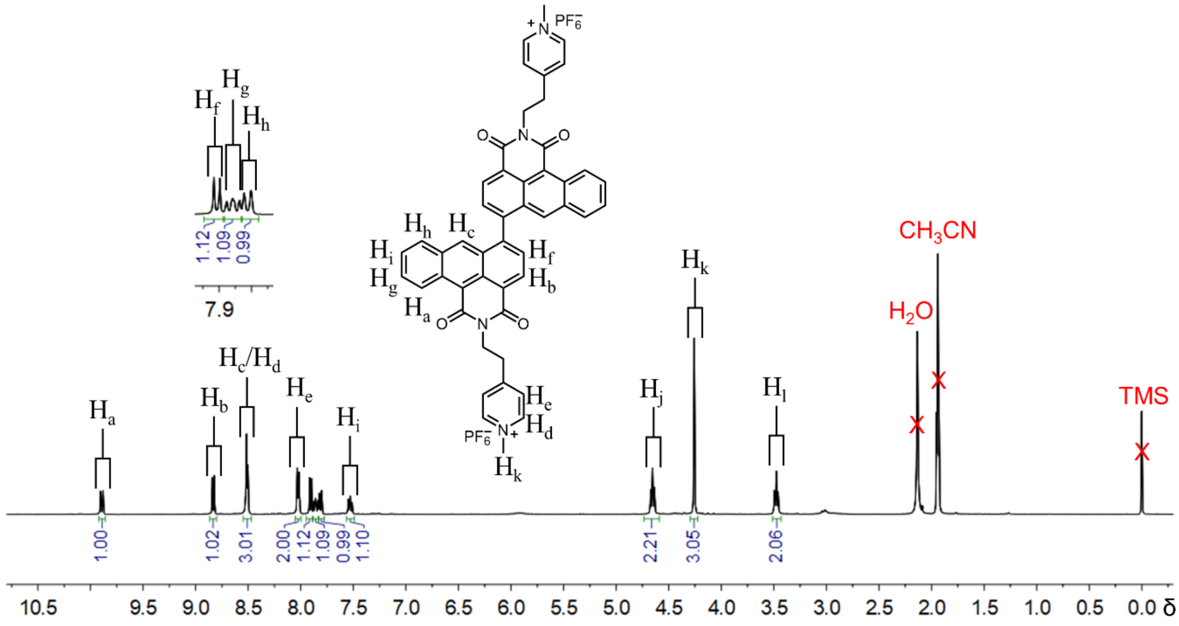

Figure S19. ^{1}H NMR spectrum of **1DiAC·PF$_6$** (400 MHz, CD_3CN, 298 K).

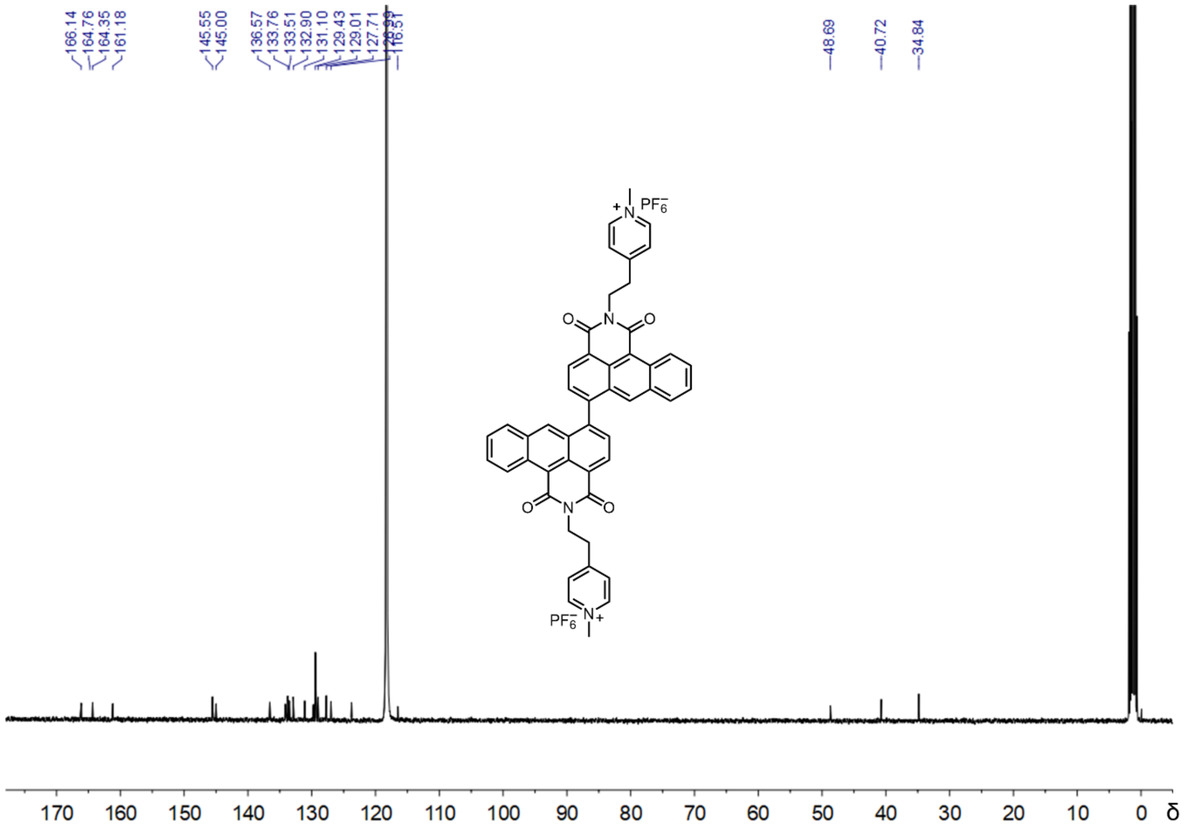

Figure S20. ^{13}C NMR Spectrum of **1DiAC·PF6** (101 MHz, CD_3CN, 298 K).

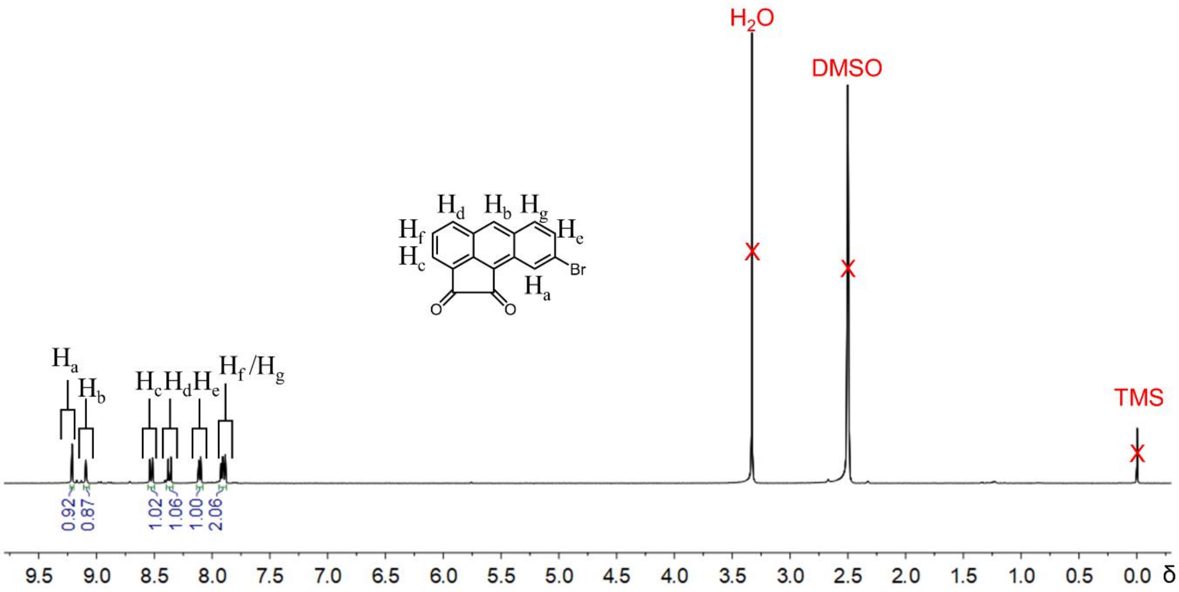

Figure S21. ^{1}H NMR spectrum of **2An Dione** (400 MHz, DMSO-d6, 298 K).

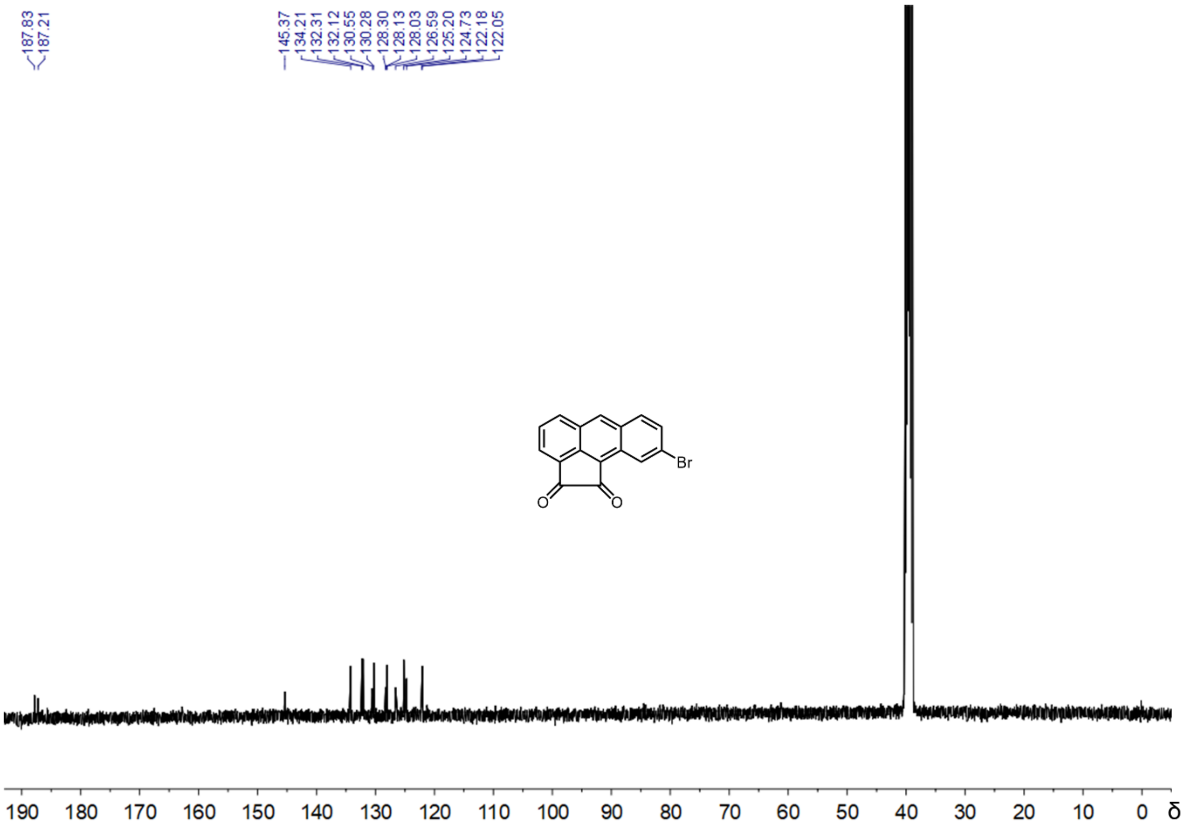

Figure S22. ^{13}C NMR Spectrum of **2An Dione** (101 MHz, DMSO-d6, 298 K).

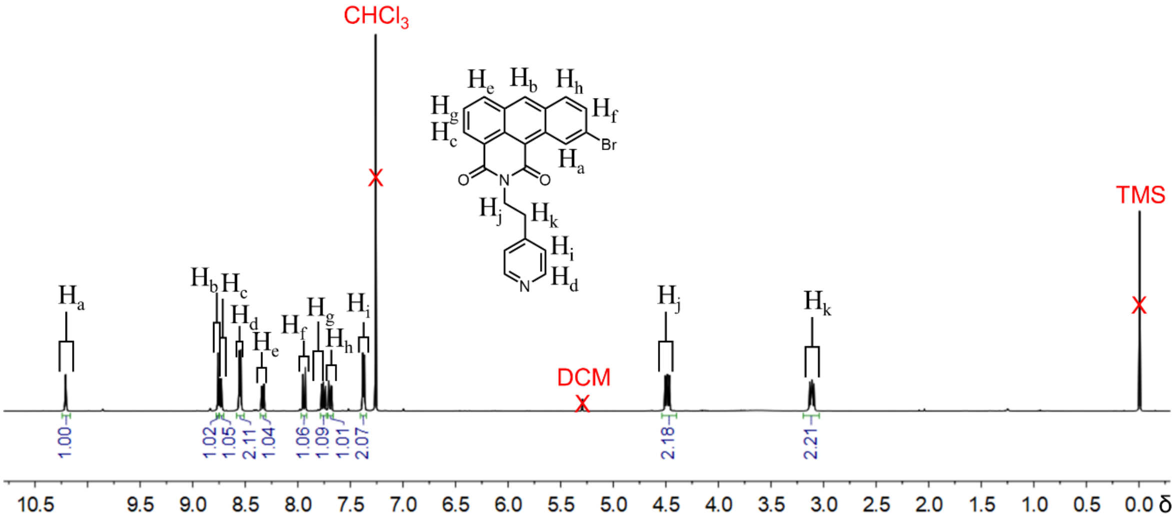

Figure S23. ^{1}H NMR spectrum of **2AC** (400 MHz, $CDCl_3$, 298 K).

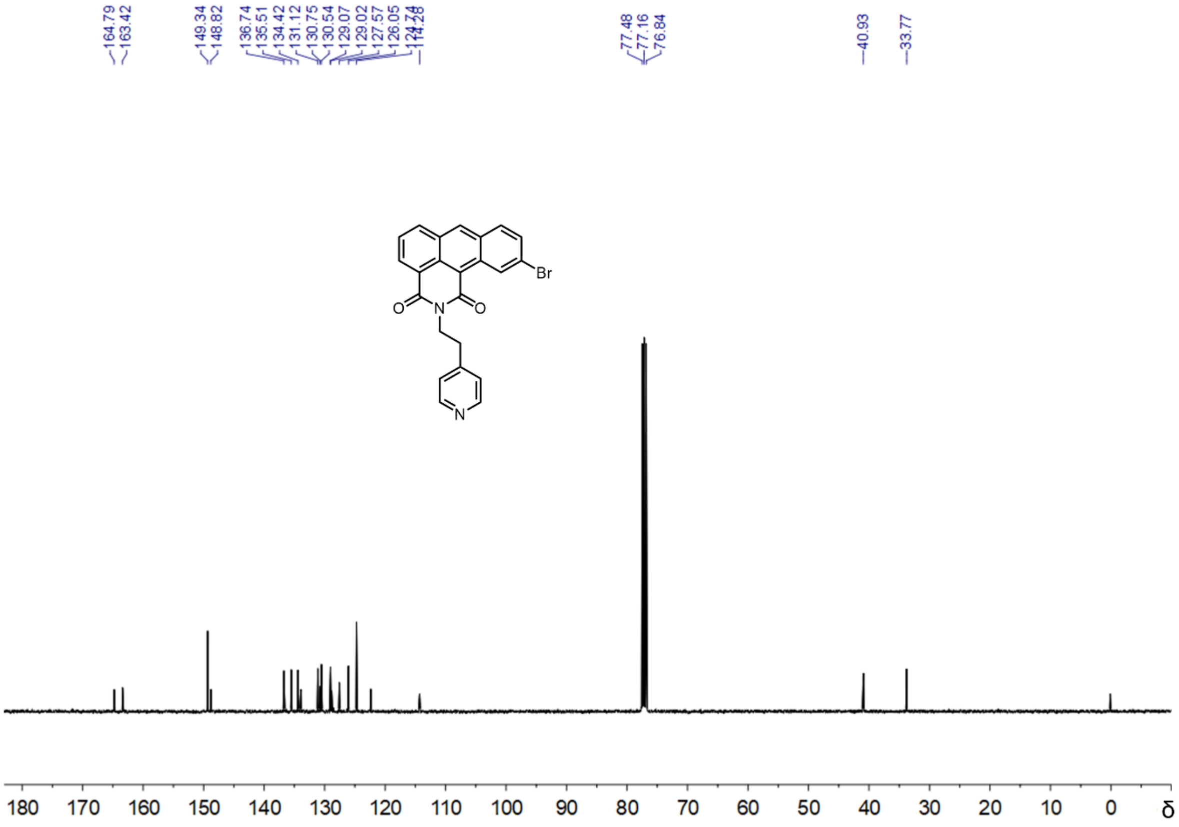

Figure S24. ^{13}C NMR Spectrum of **2AC** (101 MHz, $CDCl_3$, 298 K).

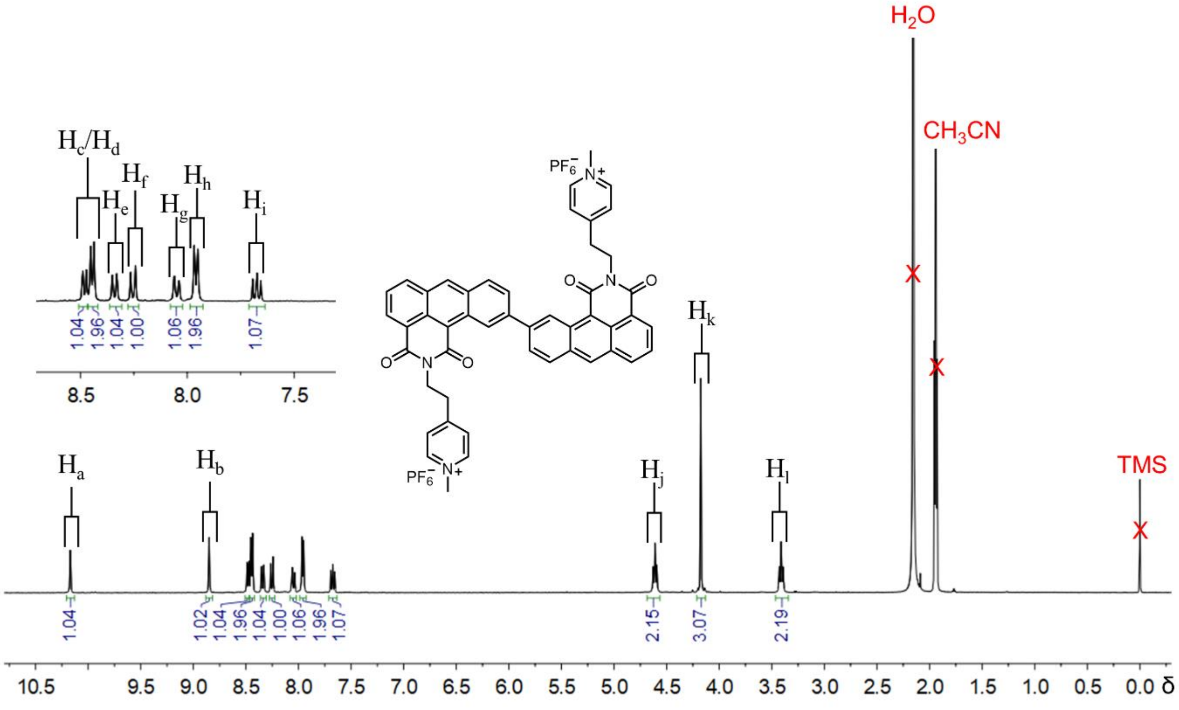

Figure S25. ^{1}H NMR spectrum of **2DiAC·PF₆** (400 MHz, CD_3CN, 298 K).

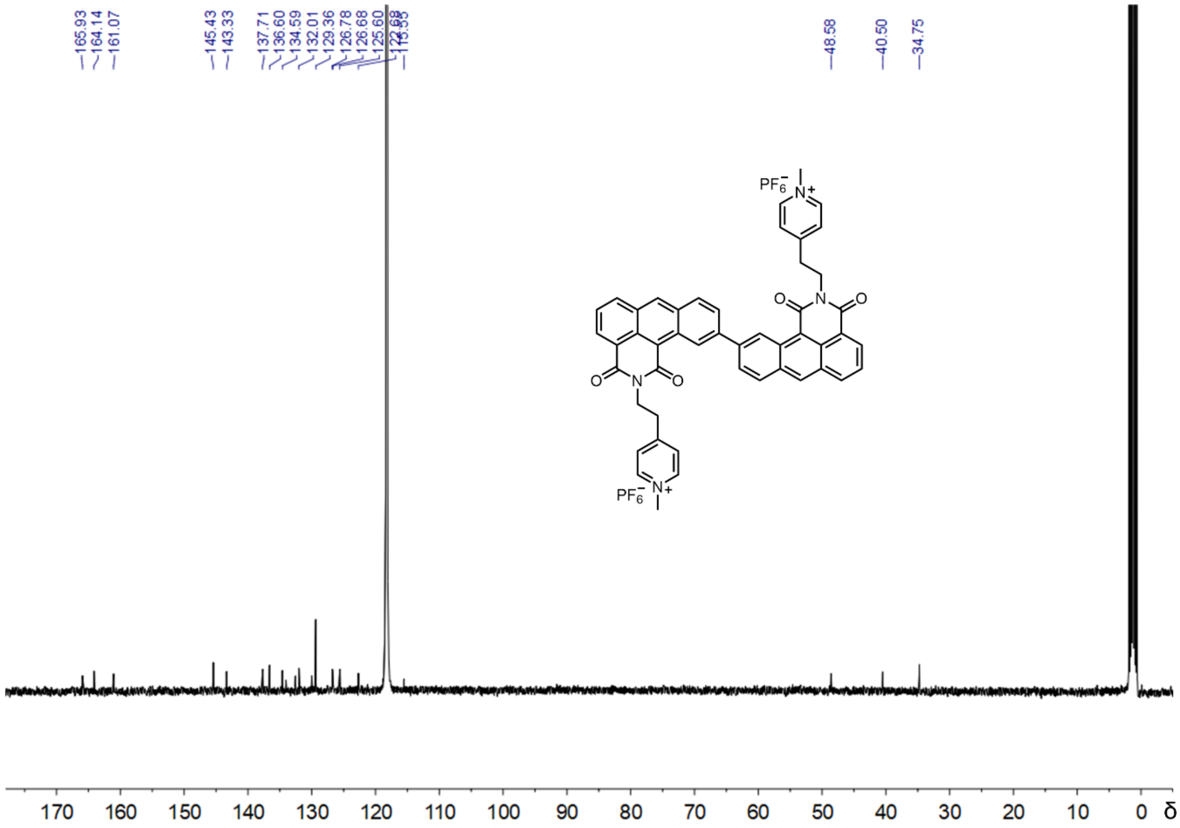

Figure S26. ^{13}C NMR Spectrum of **2DiAC·PF6** (101 MHz, CD_3CN, 298 K).

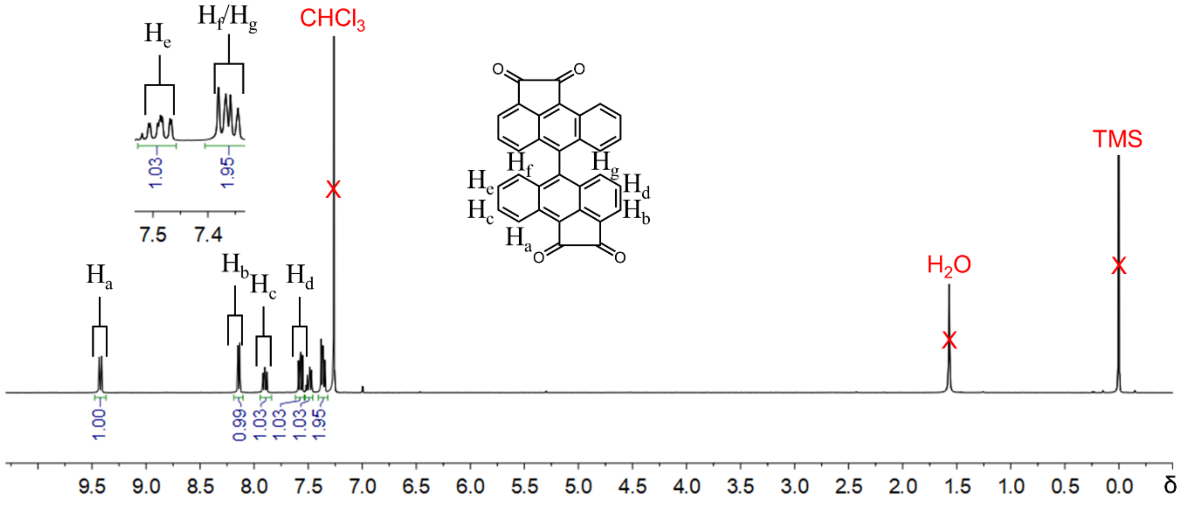

Figure S27. ^{1}H NMR spectrum of **9DiAn Dione** (400 MHz, $CDCl_3$, 298 K).

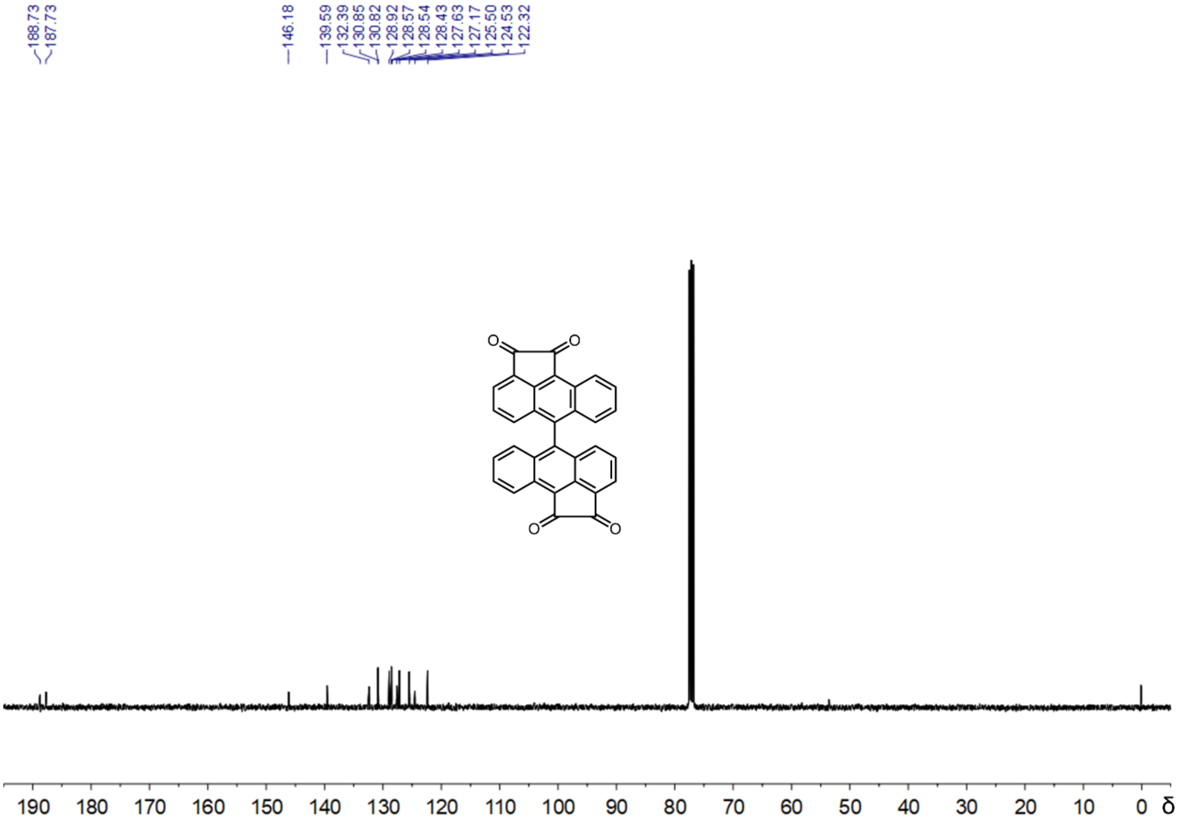

Figure S28. ^{13}C NMR Spectrum of **9DiAn Dione** (101 MHz, $CDCl_3$, 298 K).

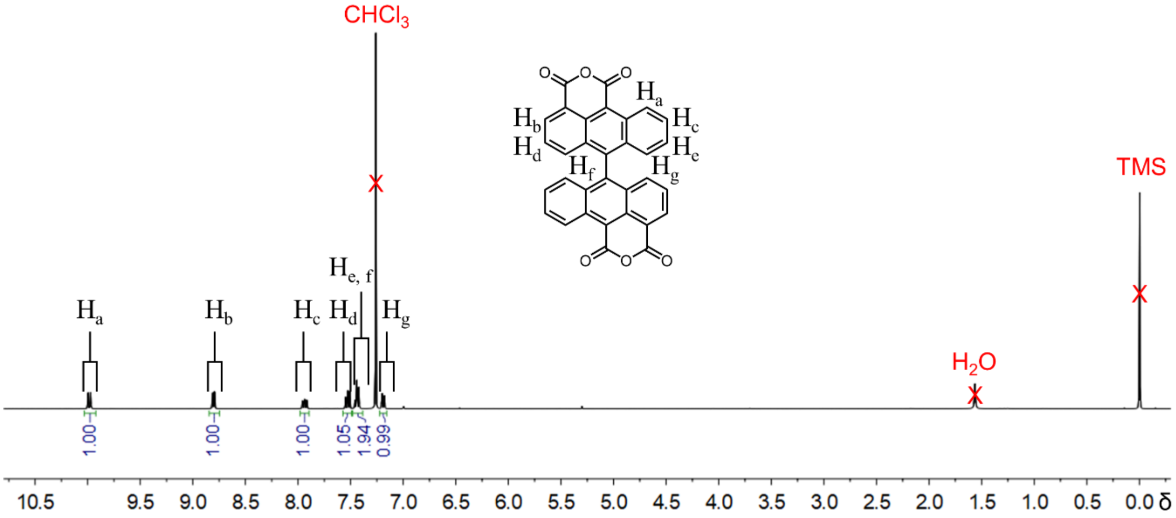

Figure S29. ^{1}H NMR spectrum of **9DiAn Anhydride** (400 MHz, $CDCl_3$, 298 K).

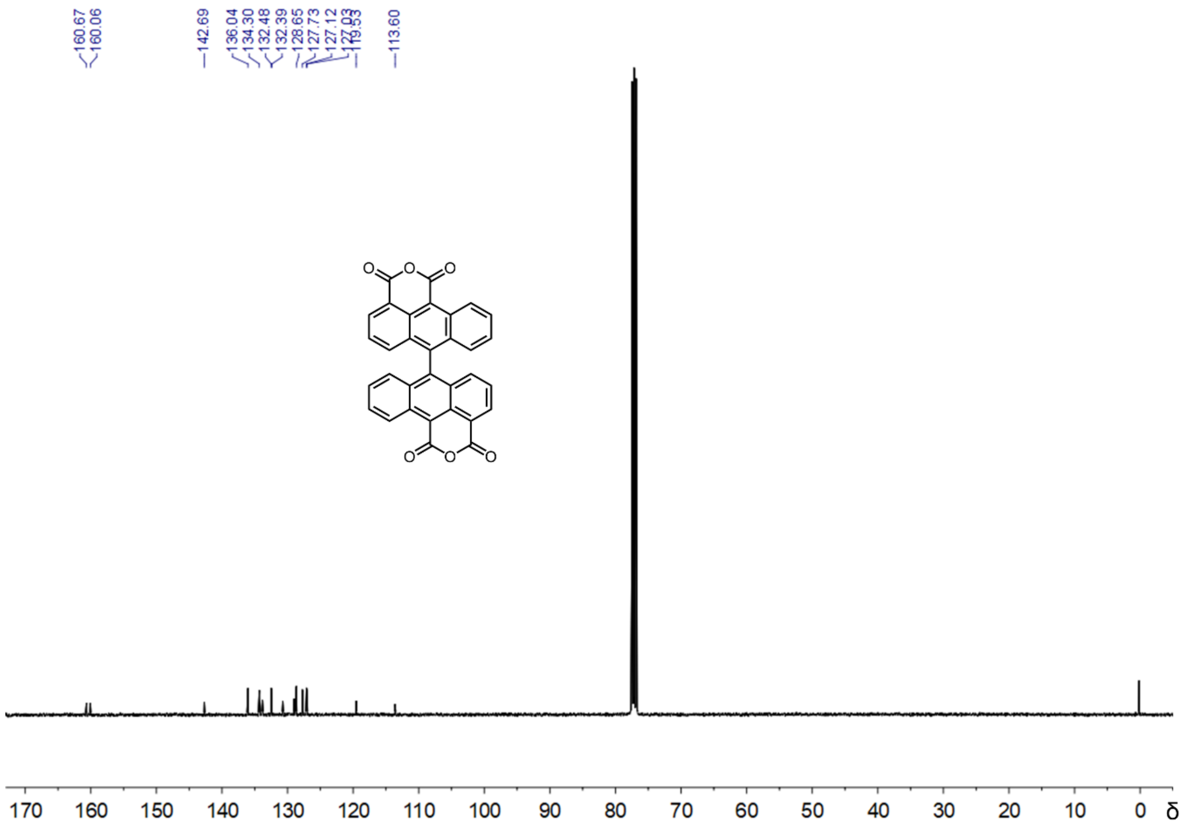

Figure S30. ^{13}C NMR Spectrum of **9DiAn Anhydride** (101 MHz, $CDCl_3$, 298 K).

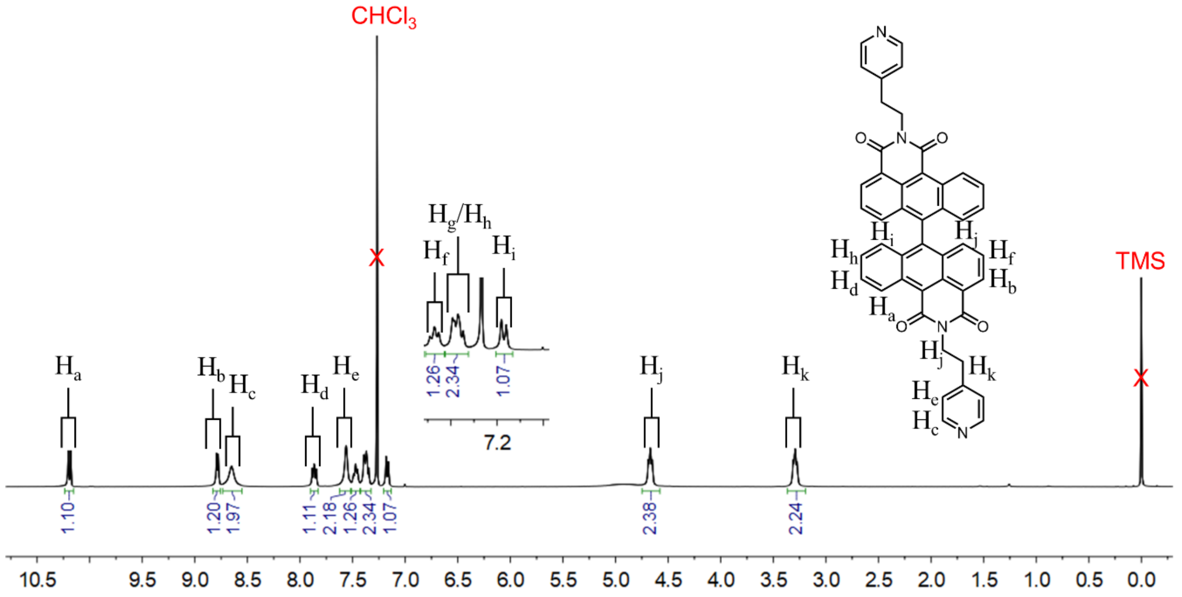

Figure S31. ^{1}H NMR spectrum of **9DiAC** (400 MHz, $CDCl_3$, 298 K).

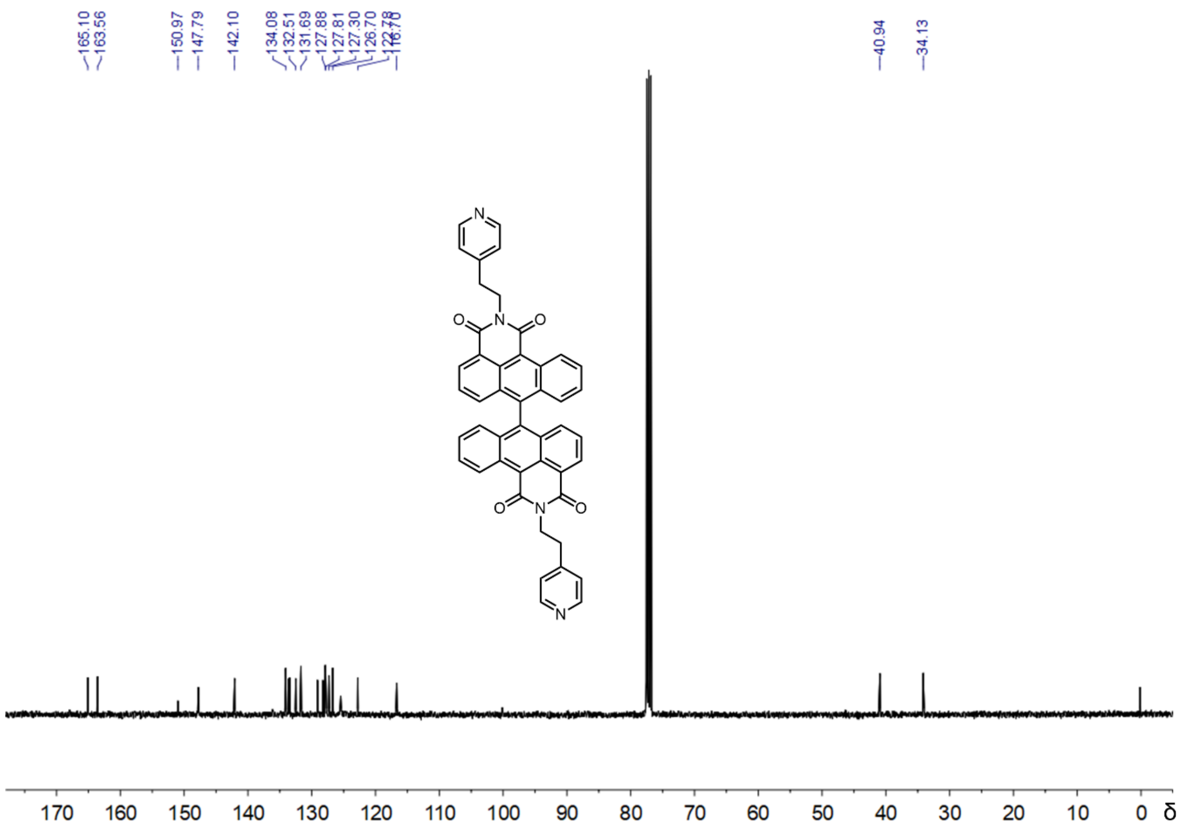

Figure S32. ^{13}C NMR Spectrum of **9DiAC** (101 MHz, $CDCl_3$, 298 K).

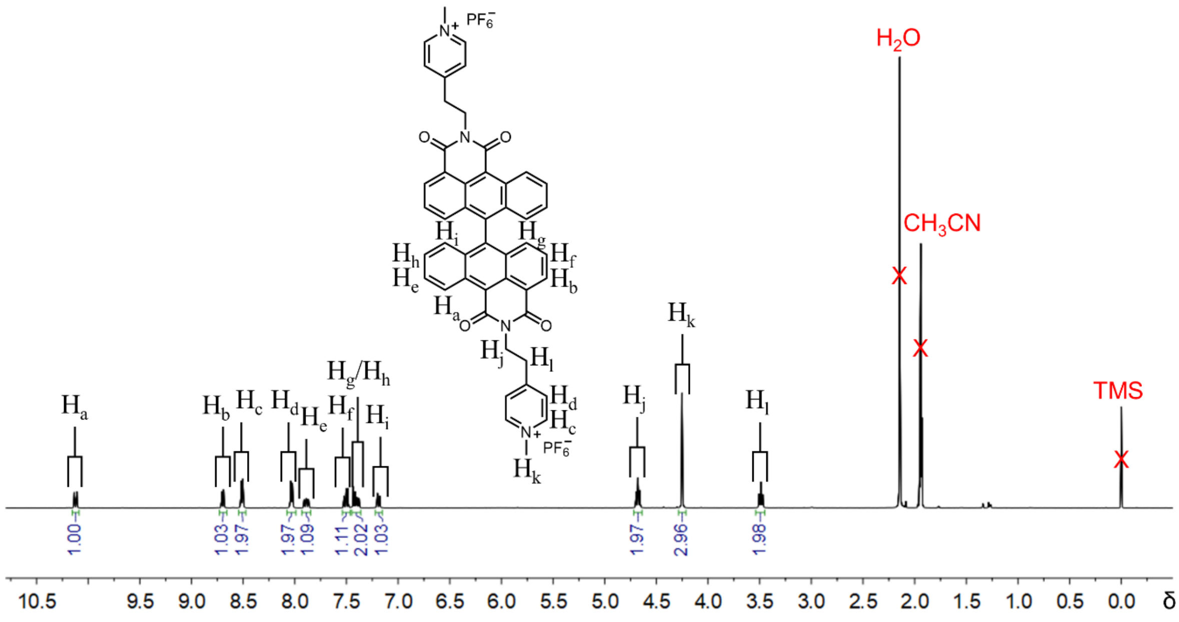

Figure S33. ^{1}H NMR spectrum of **9DiAC·PF6** (400 MHz, CD_3CN, 298 K).

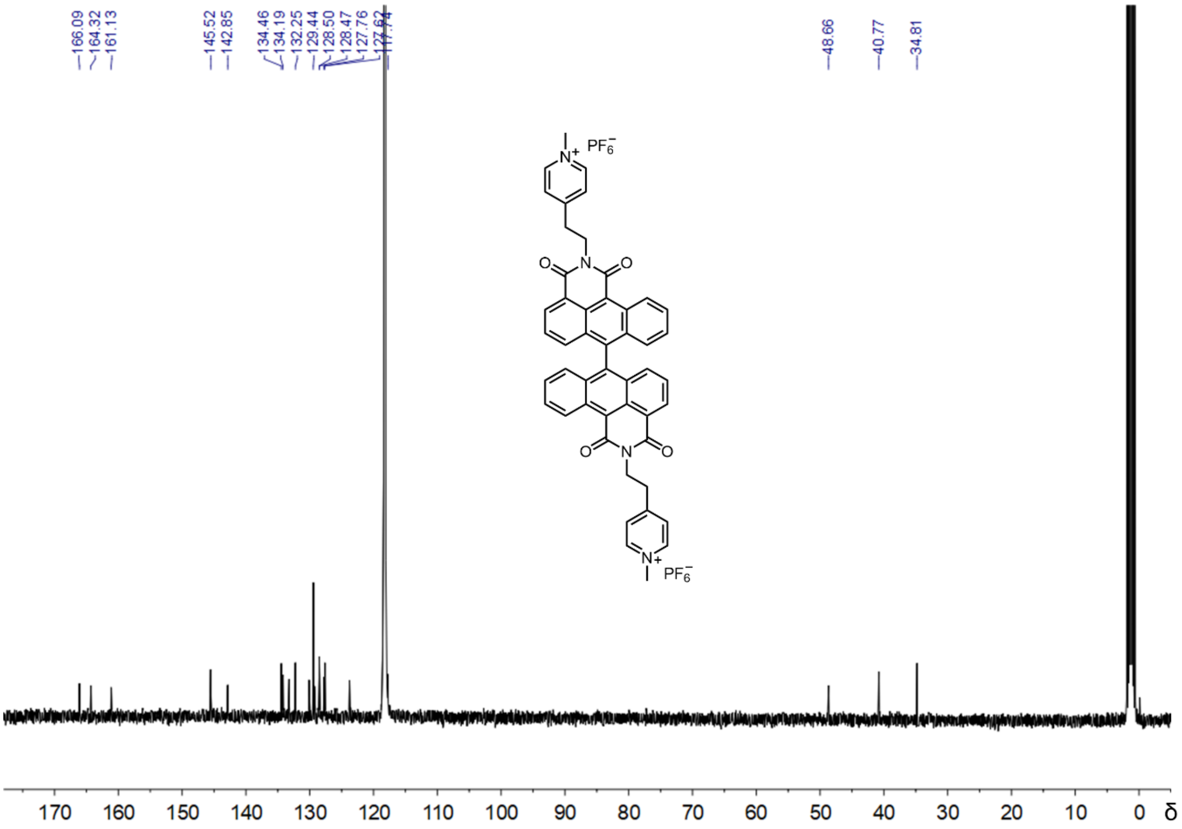

Figure S34. ^{13}C NMR Spectrum of **9DiAC·PF6** (101 MHz, CD_3CN, 298 K).

8. High Resolution Mass Spectrometry Data

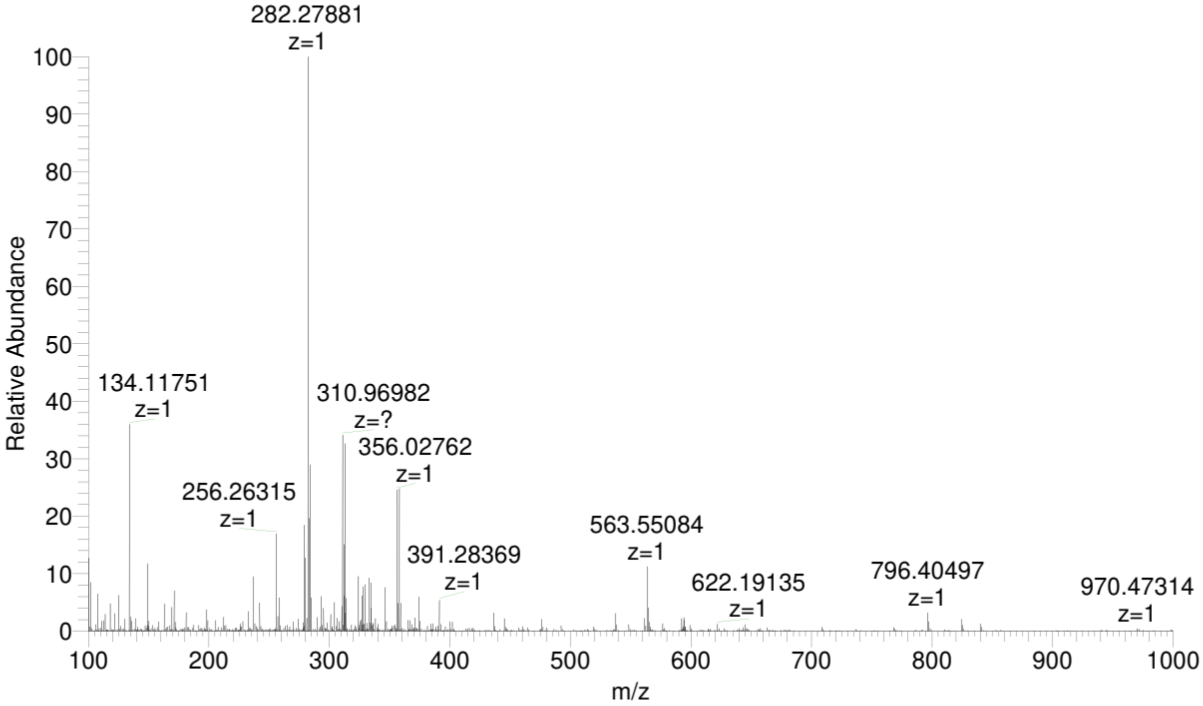

Figure S35. High resolution mass spectra of **1An Dione**.

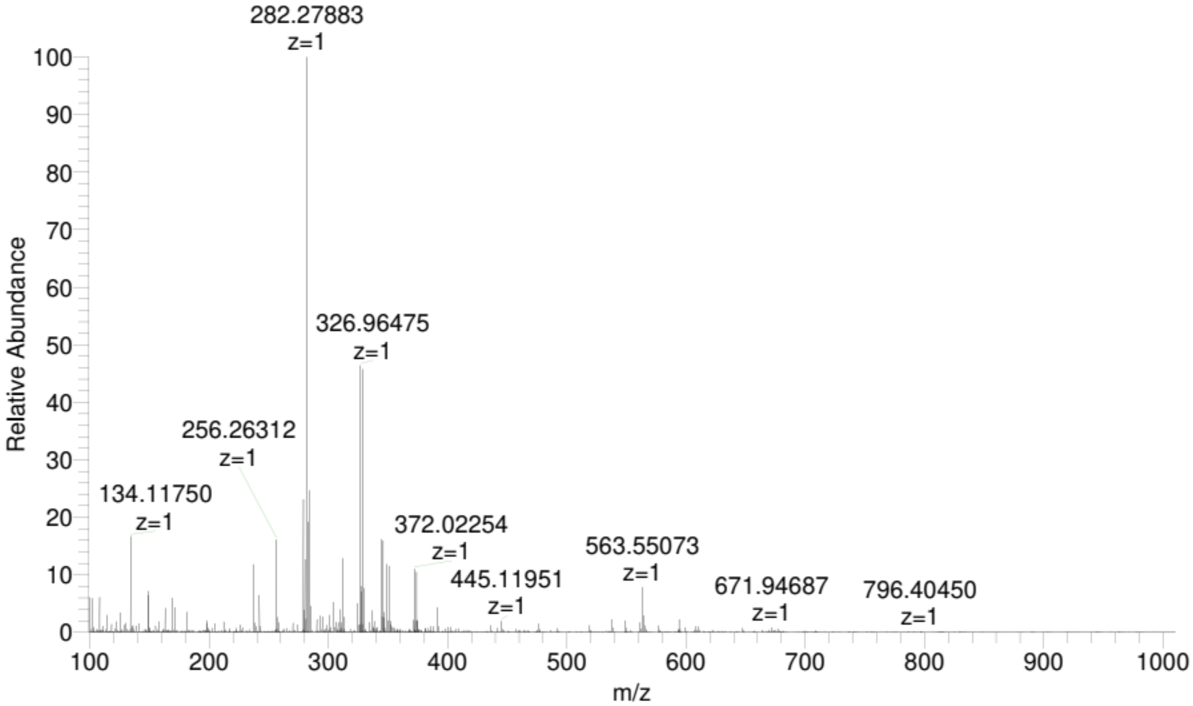

Figure S36. High resolution mass spectra of **1An Anhydride**.

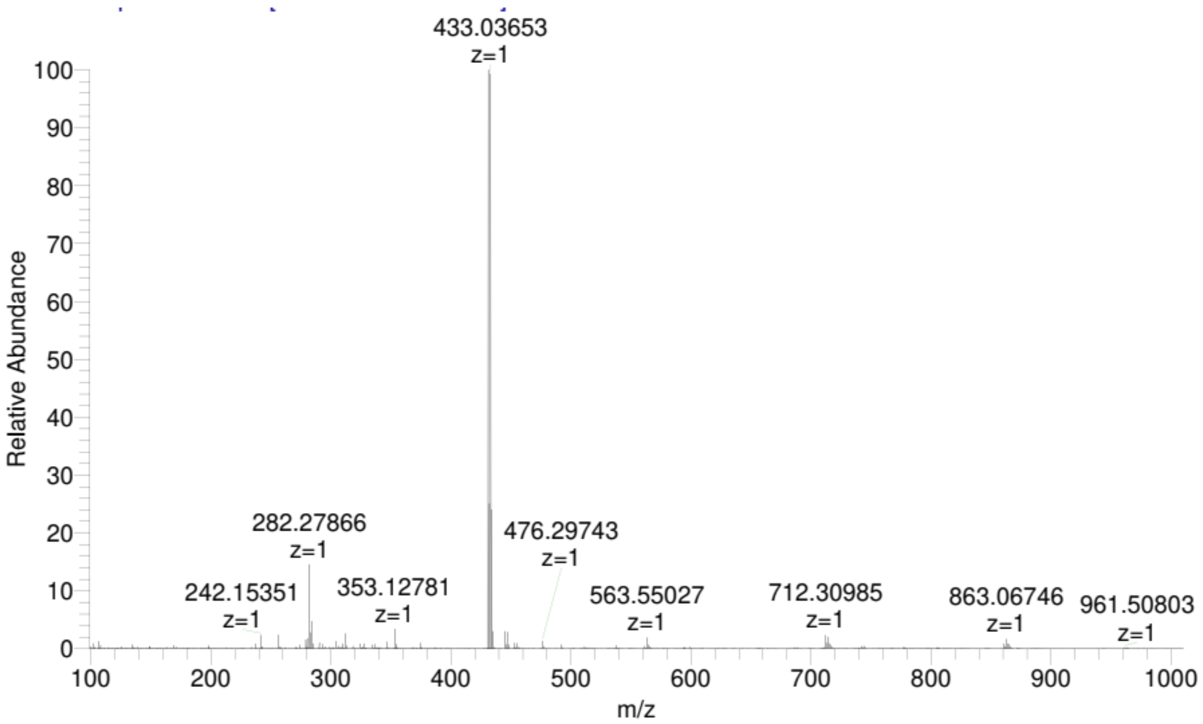

Figure S37. High resolution mass spectra of **1AC**.

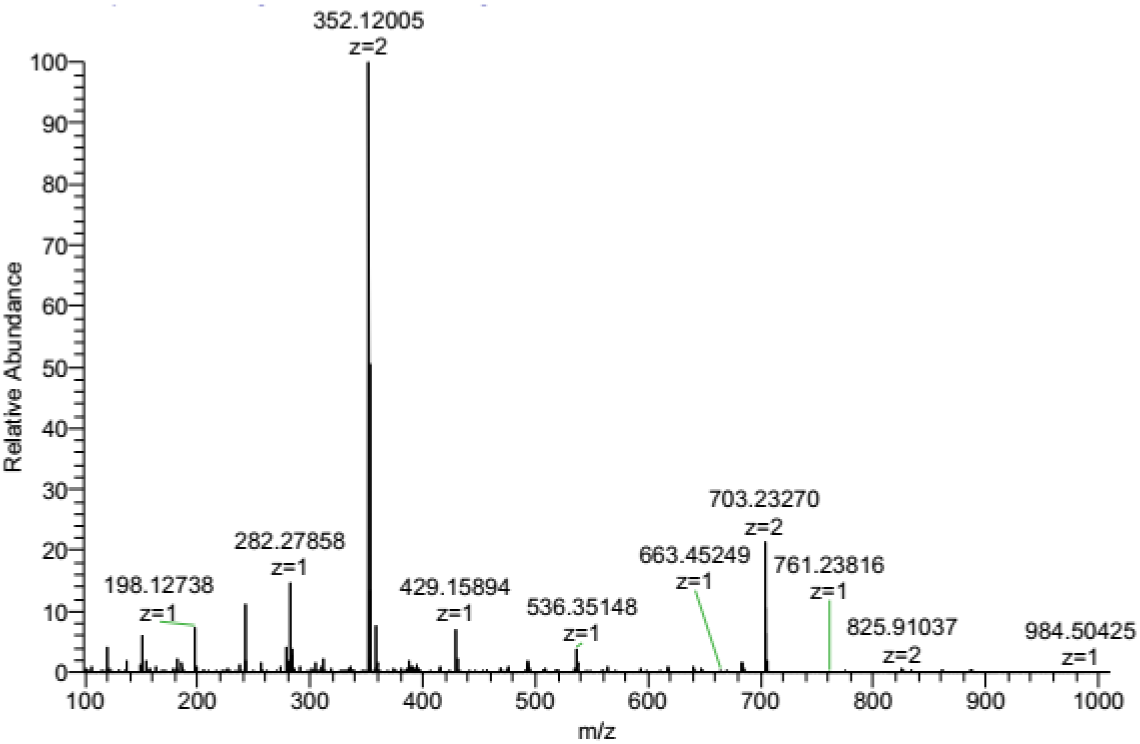

Figure S38. High resolution mass spectra of **1DiAC**.

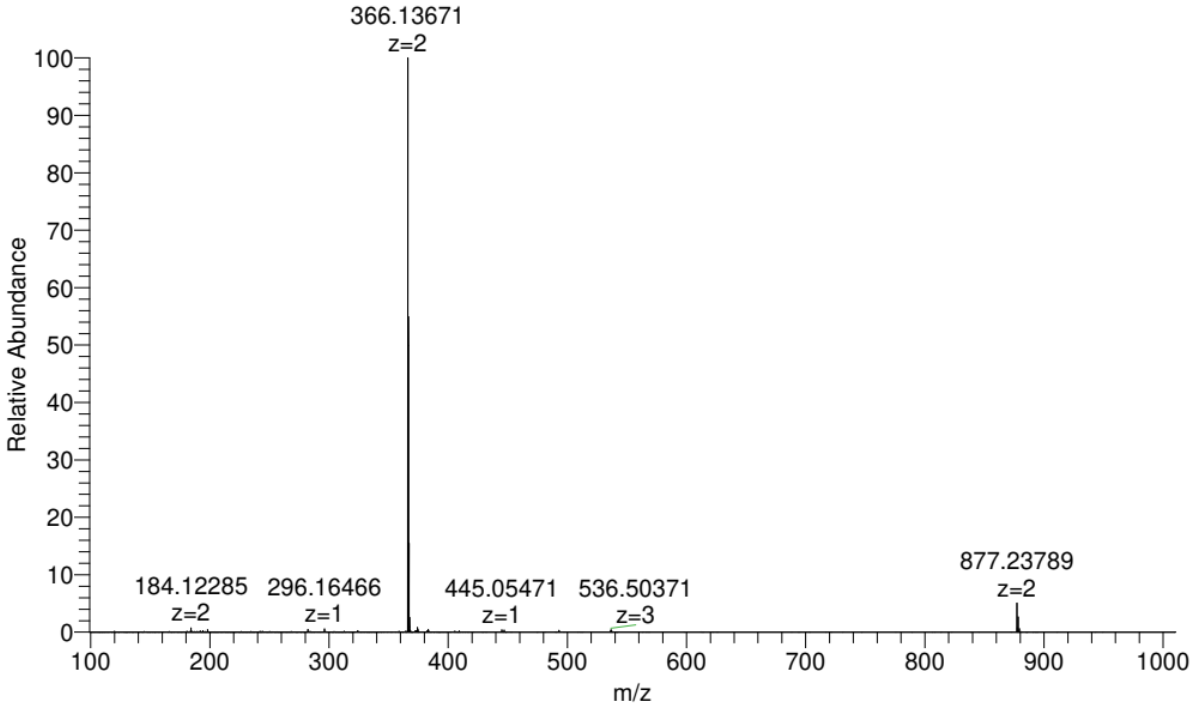

Figure S39. High resolution mass spectra of **1DiAC·PF$_6$**.

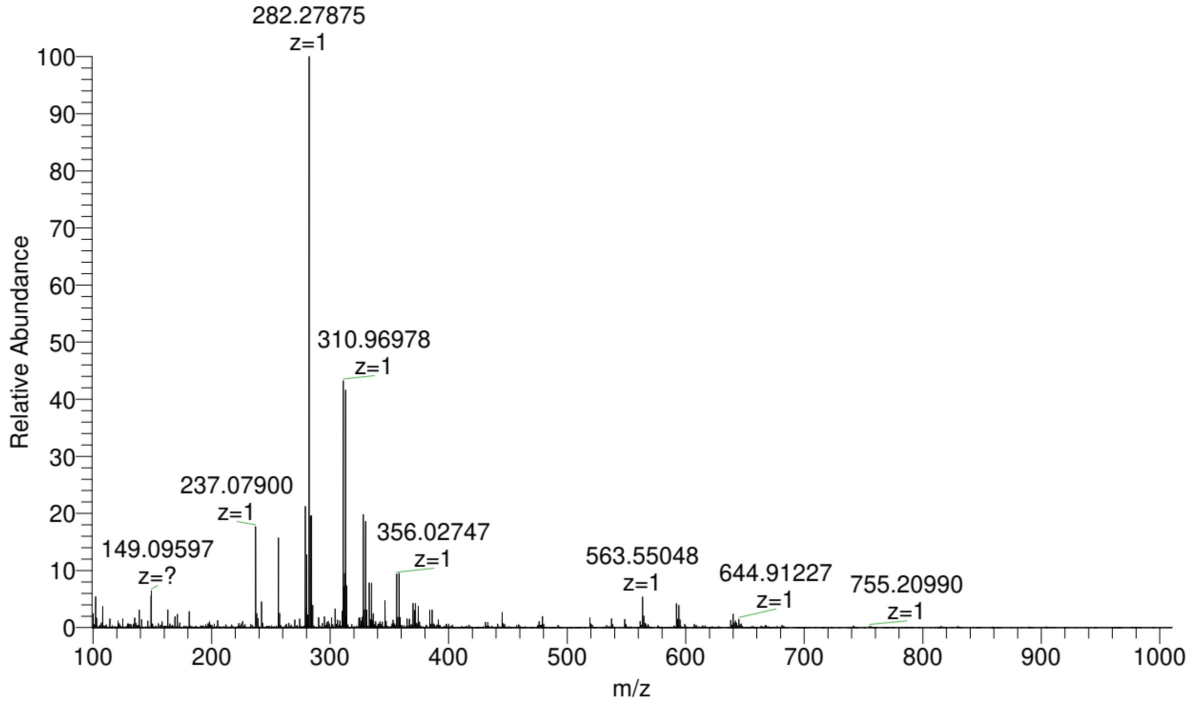

Figure S40. High resolution mass spectra of **2An Dione**.

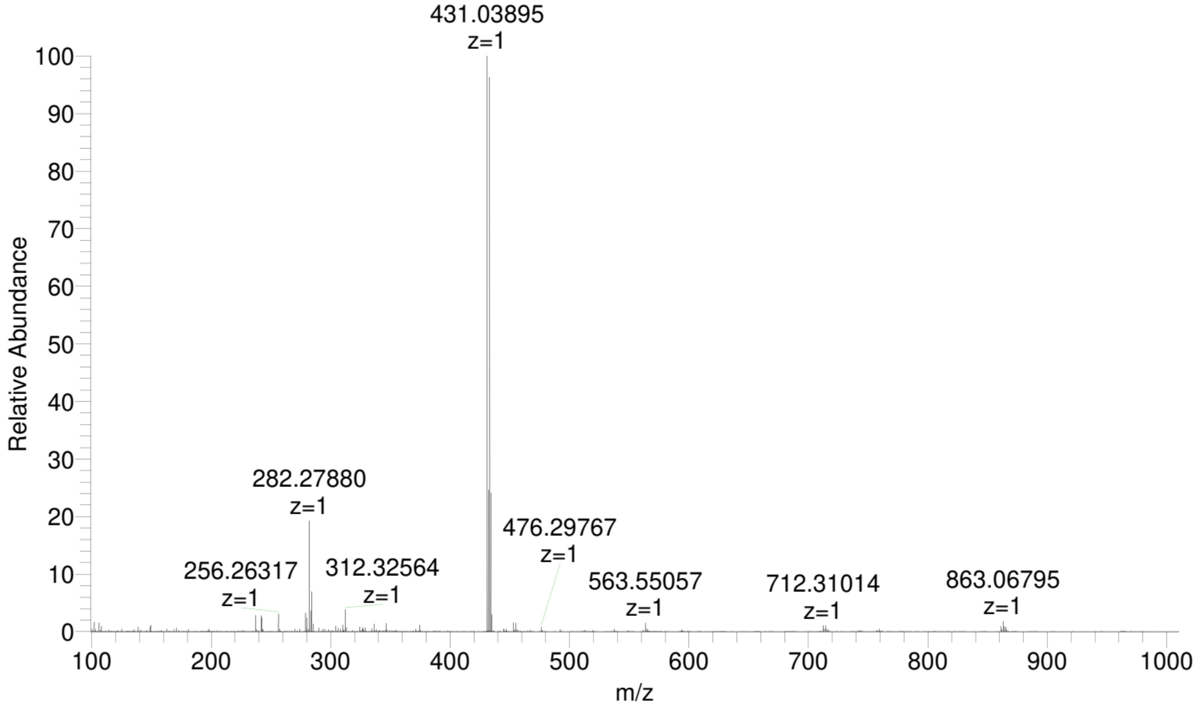

Figure S41. High resolution mass spectra of **2AC**.

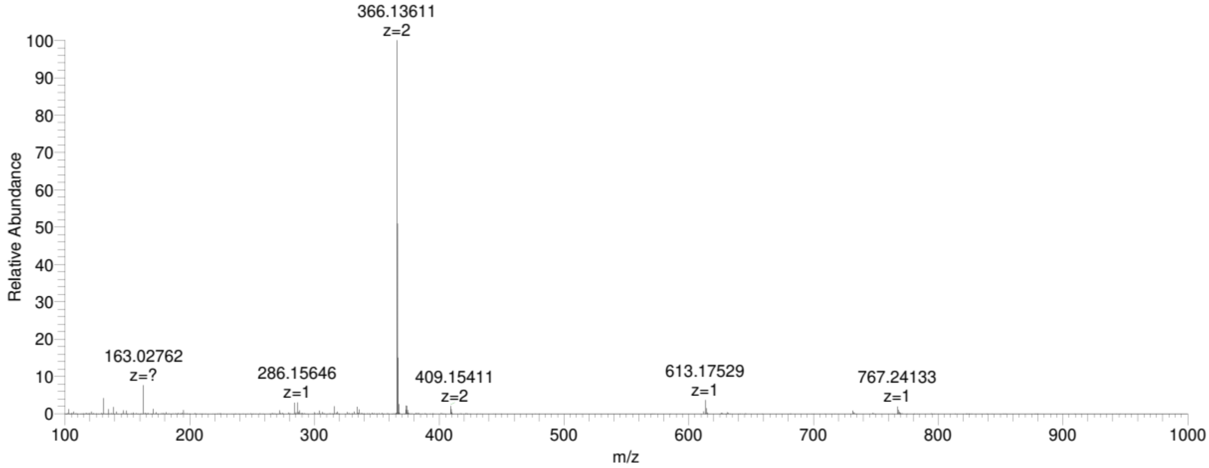

Figure S42. High resolution mass spectra of **2DiAC·PF$_6$**.

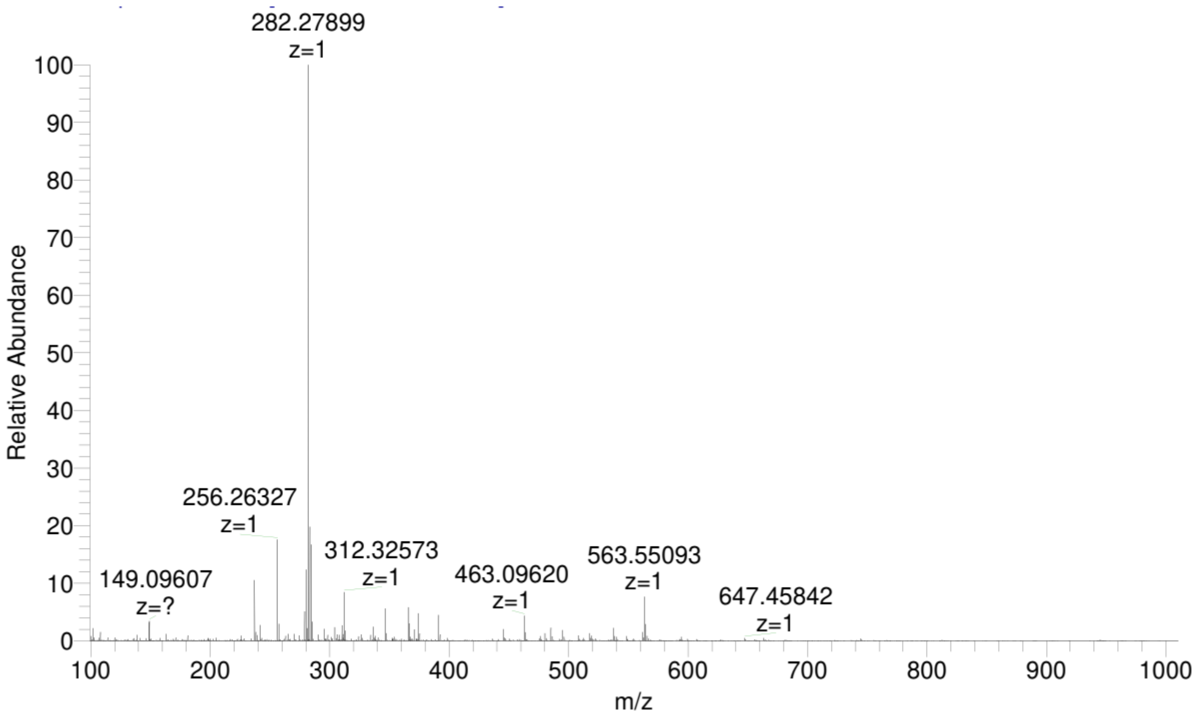

Figure S43. High resolution mass spectra of **9DiAn Dione**.

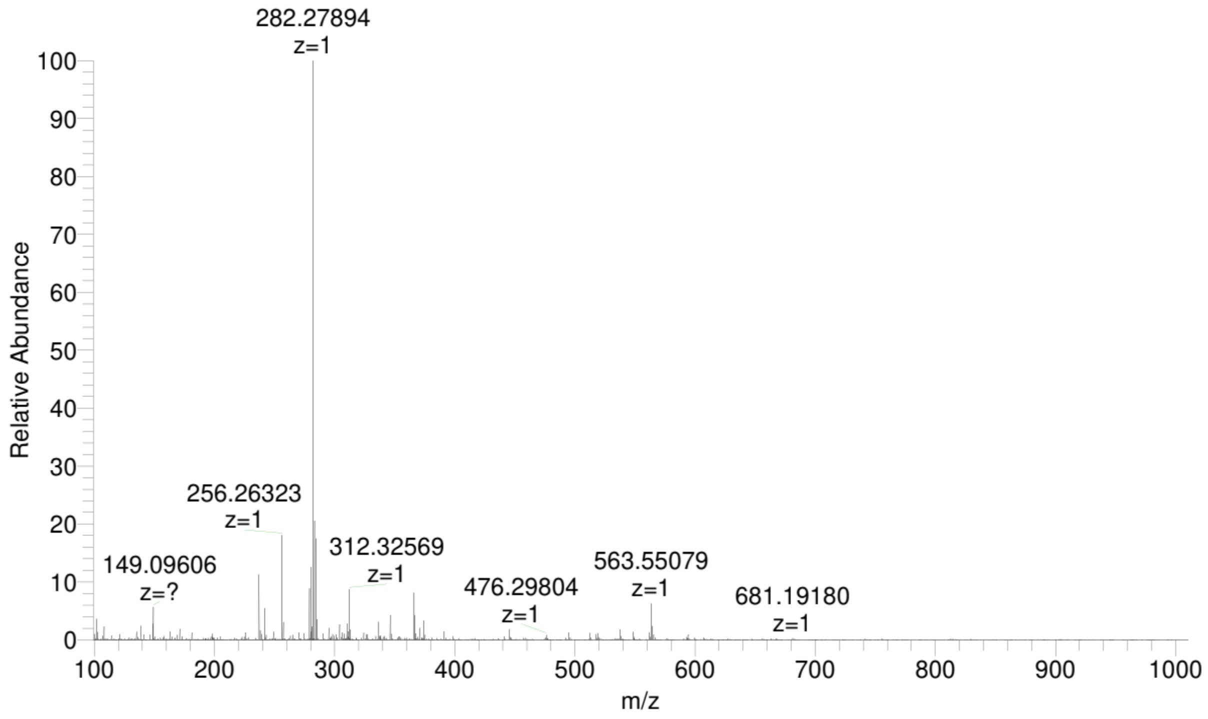

Figure S44. High resolution mass spectra of **9DiAn Anhydride**.

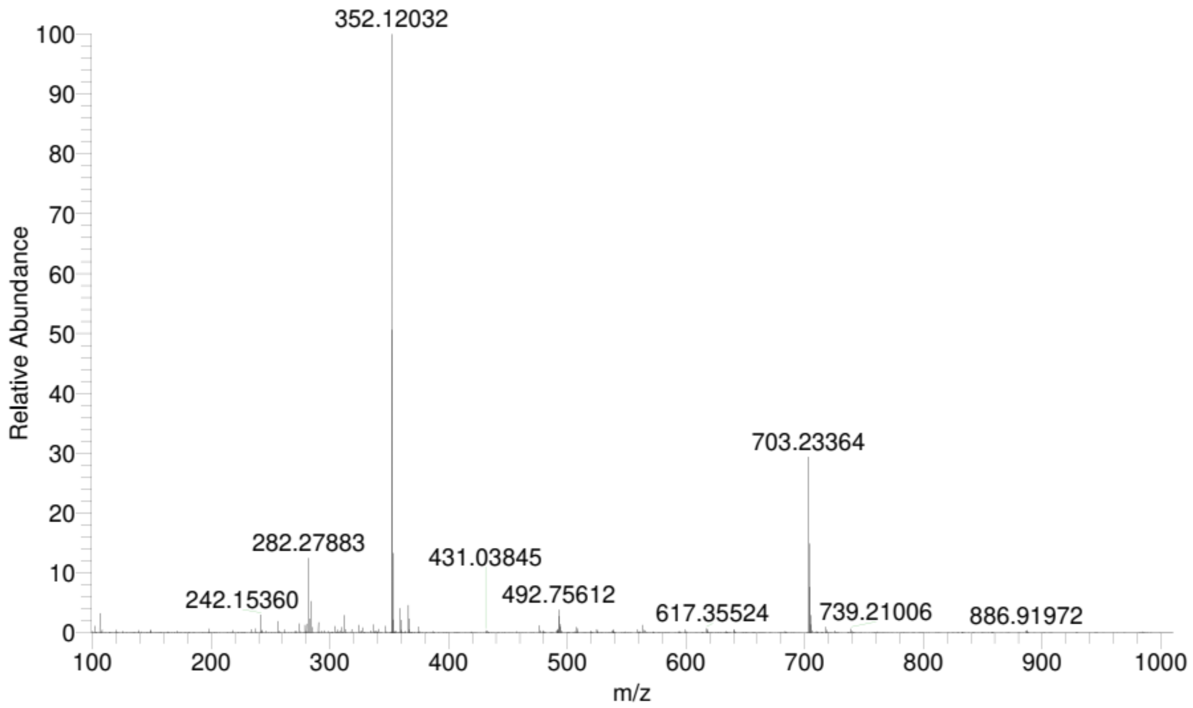

Figure S45. High resolution mass spectra of **9DiAC**.

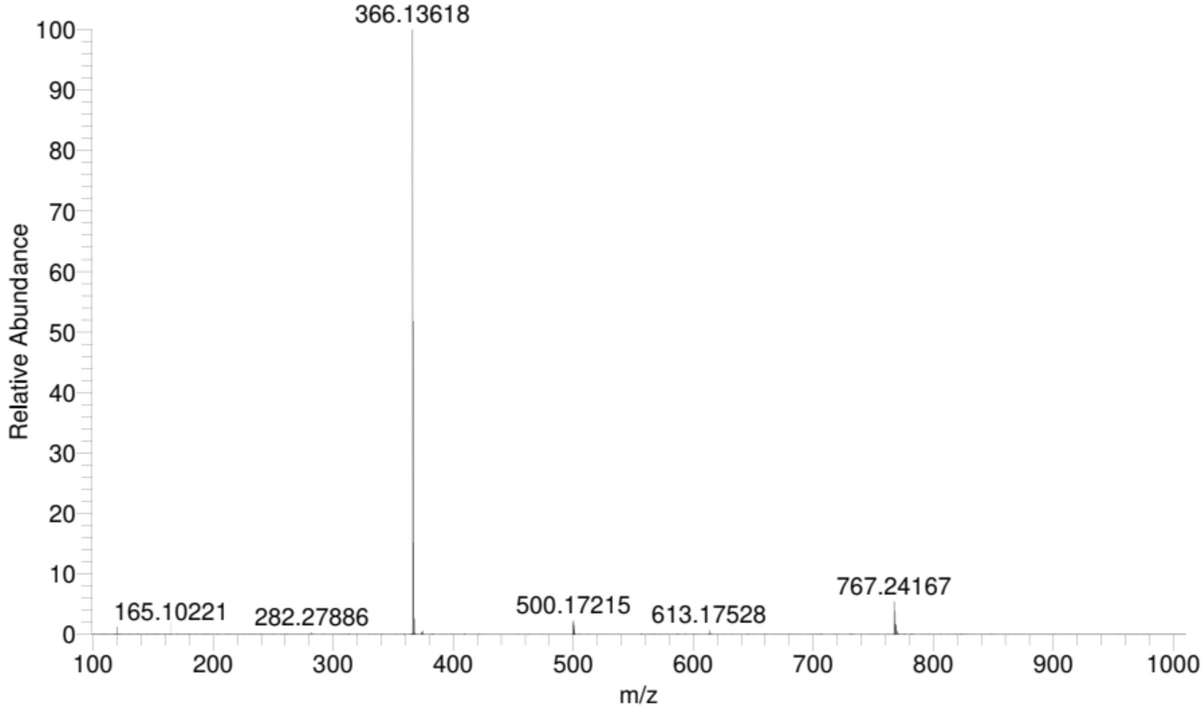

Figure S46. High resolution mass spectra of **9DiAC·PF_6**.

References

1. Dolomanov, O. V.; Bourhis, L. J.; Gildea, R. J.; Howard, J. A. K.; Puschmann, H. OLEX2: a complete structure solution, refinement and analysis program. *J. Appl. Crystallogr.* **2009,** *42*, 339-341.
2. Sheldrick, G. A short history of SHELX. *Acta Crystallographica Section A* **2008,** *64*, 112-122.
3. GaussView, Version 5, Dennington, R.; Keith, T. A.; Millam, J. M. Semichem Inc., Shawnee Mission, KS, **2016**.
4. T. Lu, F. Chen, Multiwfn: A multifunctional wavefunction analyzer. *J. Comput. Chem.* **2012**, *33*, 580-592.
